# ZeXieYin formula alleviates atherosclerosis by regulating SBAs levels through the FXR/FGF15 pathway and restoring intestinal barrier integrity

**DOI:** 10.1186/s13020-025-01116-y

**Published:** 2025-05-25

**Authors:** Shihan Zhou, Shangbo Hua, Xinyi Chen, Meiling Ni, Jing Liu, Yanqing Wang, Wanning Wu, Anni Ding, Zizhen Qin, Xinyu Yang, Xiaowei Chen, Boran Zhu, Haoxin Wu

**Affiliations:** 1 Nanjing University of Chinese Medicine, Nanjing, 210023 Jiangsu China; 2 Union Laboratory of Traditional Chinese Medicine for Brain Science and Gerontology, Nanjing University of Chinese Medicine, 138 Xianlin Road, Nanjing, 210023 People’s Republic of China; 3 Department of Hepatobiliary Surgery, Kunshan Affiliated Hospital of Nanjing University of Chinese Medicine, Kunshan, China; 4 School of Elderly Care Services and Management, Nanjing University of Chinese Medicine, Nanjing, 210023 People’s Republic of China; 5 Key Laboratory of Integrative Biomedicine for Brain Diseases at the School of Chinese Medicine, Nanjing University of Chinese Medicine, No. 138 Xianlin Road, Nanjing, 210023 China

**Keywords:** Atherosclerosis, ZeXieYin formula, Secondary bile acids, Gut microbiota, Metabolomics analysis

## Abstract

**Background:**

Atherosclerosis (AS) is the most common cardiovascular disease (CVD), despite an overall declining incidence, AS remains a leading cause of death worldwide. The ZeXieYin formula (ZXYF), one of the thirteen formulas recorded in HuangDiNeiJin, a classical book of Traditional Chinese Medicine (TCM), has previously demonstrated efficacy in reducing blood lipids and combating AS. However, the precise mechanism by which it regulates blood lipids remains unclear. Given the close correlation between bile acid metabolism and cholesterol metabolism, it is imperative to elucidate the intrinsic mechanisms through which ZXYF treats AS.

**Purpose of the research:**

This study aims to investigate the pivotal role of enterohepatic bile acid circulation in enhancing intestinal barrier function and mitigating AS by ZXYF.

**Materials and methods:**

The AS model was established by subjecting male ApoE^−/−^ mice to a high-fat diet (HFD). Moreover, to determine the impact of ZXYF on the integrity of the intestinal barrier, we quantified proinflammatory cytokines using RT-qPCR and ELISA. Additionally, we identified tight-junction proteins in the ileal tissues through IF. Finally, the intestinal flora metabolite and fecal bile acid composition were analyzed using 16S rRNA analysis, untargeted metabolomics analysis, and targeted metabolomics analysis.

**Results:**

The ZXYF significantly improved dyslipidemia and alleviated the formation of arterial plaques in AS mice. Furthermore, the administration of ZXYF resulted in a concurrent reduction in circulating lipopolysaccharide (LPS) levels and downregulation of pro-inflammatory cytokine mRNA expression in the ileum. Additionally, there was an enhancement observed in the expression of tight junction proteins within the intestinal tissue of AS mice. Further studies found that ZXYF significantly elevated the total bile acids (TBA) and total cholesterol (TC) levels in the fecal of AS mice. The untargeted and targeted metabolomic analyses further revealed that ZXYF exerts regulatory effects on bile acid phenotype by decreasing secondary bile acids (SBAs) levels through modulation of gut microbiota composition, such as enrichment of *Akkermansia* (AKK) abundance, and inhibition of enterohepatic circulation of bile acids. ZXYF specifically increased the expression of hepatic bile acid synthesis enzymes CYP7A1 by modulating the FXR/FGF15 signaling pathway, thereby promoting enhanced de novo bile acid synthesis and facilitating cholesterol catabolic excretion.

**Conclusion:**

The findings of our research indicate that ZXYF exerts a defensive role in the advancement of AS. The mechanism underlying the role of ZXYF in combating AS is closely associated with gut microbiota reshaping and regulation of enterohepatic bile acid circulation.

**Graphical Abstract:**

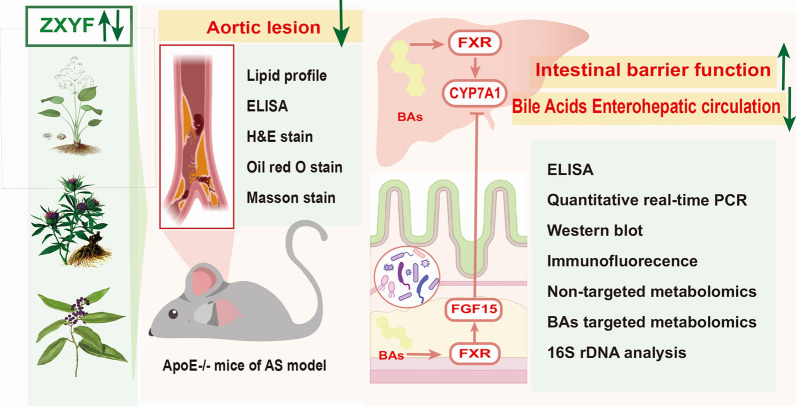

**Supplementary Information:**

The online version contains supplementary material available at 10.1186/s13020-025-01116-y.

## Introduction

The most prevalent type of cardiovascular disease is Atherosclerosis (AS), which is intimately associated with chronic inflammation of the blood vessels and dysregulation of lipid metabolism, leading to the formation of atherosclerotic plaques and narrowing of the vessels. Importantly, AS is considered the underlying cause of various cardiovascular and peripheral vascular diseases [1]. Recently, numerous studies have indicated a potential correlation between the cardiovascular system and gastrointestinal homeostasis, suggesting that the severity of AS is directly proportional to intestinal permeability [2]. The intestinal barrier system, which is primarily constituted by microbial, chemical, physical, and immunological components, functions as the frontline defense mechanism against foodborne antigens. Dysregulation of the gut barrier integrity can result in disrupted energy metabolism and immunological homeostasis, thereby contributing to perturbations in lipid metabolism and vascular inflammatory responses. These multifactorial pathophysiological cascades ultimately give rise to the progression of AS [3]. Current therapeutic strategies for AS, including lipid-lowering medications and anti-inflammatory agents such as statins and proprotein convertase subtilisin/kexin type 9 (PCSK9) inhibitors, demonstrate limited therapeutic efficacy in preventing AS initiation and progression. Prolonged administration of these agents is associated with significant adversarial effects, encompassing muscle-related adversities (myalgia, rhabdomyolysis) and hepatotoxicity [4]. These limitations underscore the critical need for developing novel therapeutic paradigms with enhanced efficacy and improved safety profiles. Previous research findings have demonstrated significant alterations in bile acid levels among patients with AS. Bile acids actively participate in cholesterol metabolism and contribute to the protection of the intestinal barrier, thus regulating bile acid metabolism is considered a reliable approach for treating AS [5]. The ATP binding cassette subfamily G members 5 and 8 (ABCG5/8) play a crucial role in the elimination of cholesterol from both the liver and intestines. Simultaneously, primary bile acids such as cholic acid (CA) and chenodeoxycholic acid (CDCA) are synthesized through hepatic metabolism of cholesterol via the key enzyme Cholesterol 7α-hydroxylase (CYP7A1) conversion. These primary bile acids react with glycine or taurine to form conjugated bile acid, thereby augmenting their aqueous solubility. Then, bile acids are released from the gallbladder into the duodenum and small intestine, where they facilitate the emulsification of fats, breaking them down into minute fat droplets, thereby increasing the surface area for enzymatic action and promoting the absorption of fats and fat-soluble vitamins [6].

The enterohepatic circulation of bile acids, a critical pathway for bile acid reabsorption and recycling, serves as a fundamental regulator of cholesterol homeostasis. Approximately 95% of intestinal bile acids undergo efficient reabsorption in the distal ileum through active transport mechanisms in enterocytes, subsequently returning to the liver via portal circulation [7]. The remaining 5% of bile acids that escape ileal absorption progress to the colon, where gut microbiota mediate their biotransformation into secondary bile acids (SBAs), predominantly deoxycholic acid (DCA) and lithocholic acid (LCA). These microbially modified SBAs demonstrate differential metabolic fates: a portion undergoes enterohepatic recirculation through colonic reabsorption, while the residual fraction undergoes fecal excretion. Notably, the nuclear receptor farnesoid X receptor (FXR/NR1H4) and its downstream effector fibroblast growth factor-15/19 (FGF15/19) constitute a pivotal regulatory axis governing intestinal-hepatic bile acid metabolism. Emerging evidence suggests that tissue-specific FXR activation modulates bile acid synthesis through negative feedback mechanisms, maintaining systemic bile acid homeostasis [8].

The distinguishing features of Traditional Chinese Medicine (TCM) lie in its minimal withdrawal symptoms and its capacity to address a multitude of health concerns. ZeXieYin formula (ZXYF), which consisted of Zexie (*Alisma plantago-aquatica* L.), Baizhu (*Atractylodis macrocephala* Koidz*.*), and Luxinacao (*Pyrola calliantha* Andres.) at a ratio of 2:2:1. It was originally documented in HuangdiNeijing, which is one of the most significant works in Chinese medicine. In TCM, ZXYF is regarded as having the effects of tonifying the spleen (Jian-Pi), resolving dampness (Hua-Shi), and clearing heat (Qing-Re). It is commonly used to treat syndromes such as spleen deficiency with excessive dampness and damp-heat mutual constraint, which highly aligns with the pathogenesis of AS. In clinical observations, the prescriptions combining *Rhizoma Alismatis* and *Rhizoma Atractylodis* Macrocephalae (such as Wuling Powder and Zexie Decoction) can increase the blood lipid levels of patients with phlegm-dampness type hyperlipidemia. Meanwhile, our previous animal studies have shown that ZXYF has the potential to be a therapeutic intervention for AS through its anti-inflammatory properties [9], modulation of gut microbiota composition [10], and promotion of macrophage transformation [11]. It is imperative to emphasize that dysregulated cholesterol metabolism constitutes a significant risk factor for AS. Our previous research has demonstrated the efficacy of ZXYF in reducing circulating cholesterol levels. However, the underlying mechanisms of ZXYF in improving hyperlipidemia and preventing AS remain uncertain, particularly regarding its impact on the FXR/FGF15-regulated enterohepatic circulation of bile acids and cholesterol metabolism.

In our study, the AS model was established in ApoE^−/−^ mice by feeding HFD, while Atorvastatin served as the positive control drug to assess the lipid regulatory effects of ZXYF. The subsequent application of untargeted and targeted metabolomic analyses was employed to further investigate the regulatory effects of ZXYF on bile acid metabolism. Apart from that, 16S rRNA analysis and an in vitro experiment were conducted to validate the potential of ZXYF in attenuating lipid accumulation and preventing atherosclerosis by activating FXR signaling pathway while inhibiting enterohepatic bile acid circulation.

## Materials and method

### Drugs preparation

The composition of ZXYF was depicted in Table S1, based on our previous research findings [7]. Zexie (150 g), Baizhu (150 g), and Luxiancao (75 g) were procured from the Outpatient Department of National Medical Hall at Nanjing University of Traditional Chinese Medicine in Nanjing, China. These herbs were accurately weighed and soaked in 2 L of water for one hour. After a boiling period of 40 min, the solution was filtered through a double layer of gauze. Subsequently, the residue was mixed with an additional liter of water and decocted under vacuum pressure for half an hour. The resulting filtrates were combined and suspended in 400 mL volume. Further concentration to achieve concentrations of 0.5 g/mL and 1 g/mL was accomplished using rotary evaporation at a constant temperature of 55 °C. Finally, the ZXYF liquid was stored at − 20 °C for future use. HPLC and HPLC–MS techniques were employed to identify the primary constituents, while Table S2 presented the fingerprint analysis results along with Figure S1 showcasing the main components.

### Animals and experimental design

**Fig. 1:**
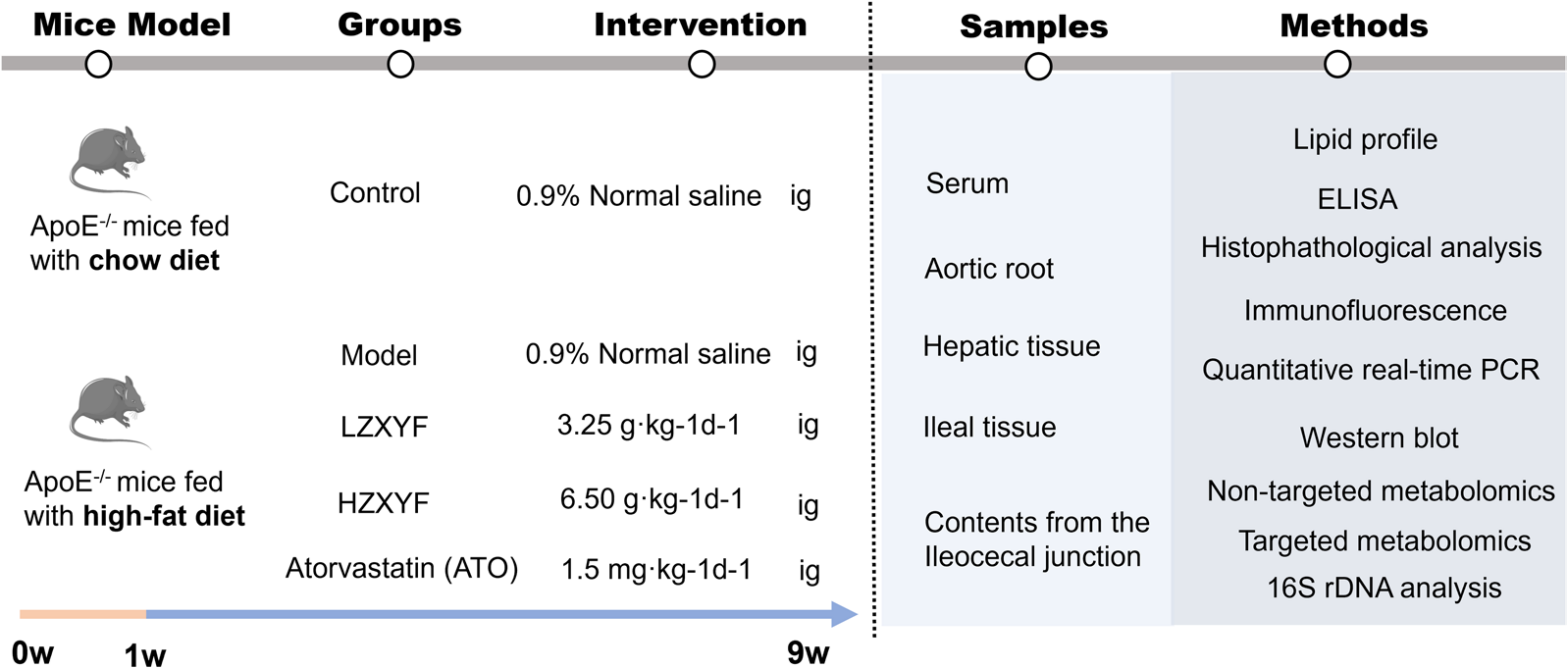
The flow diagram of the experimental design

We obtained 36 male APOE^−/−^ specimens, aged between 6 and 8 weeks, weighing approximately 20 ± 5 g, from Changzhou Cavens Laboratory Animal Sino Co. Ltd (License NO. SCXK (SU)-2016–0010). The experimental animals were granted approval by the Ethics Committee for Animal Protection of Nanjing University of Chinese Medicine (Approval No. 202209A066). The mice were housed in a specific pathogen-free (SPF) environment maintained at a temperature of 18–22 °C with humidity levels ranging from 50 to 70%, following a light/dark cycle of 12 h each. The mice had ad libitum access to food and water. The animal experiments were conducted in strict adherence to ethical standards for animal care and followed a double-blind protocol. Each test involving animals strictly adhered to the ethical standards for their care, and all experiments were carried out in a double-blind fashion across five groups: the control group (CON, *n* = 6), the model group (MOD, *n* = 6), t The mice were divided into four groups: the high-dose ZXYF drink group (HZXYF, *n* = 6), the low-dose ZXYF drink group (LZXYF, *n* = 6), the atorvastatin gavage group (ATO, *n* = 6), and the control group (CON) which was fed a regular chow diet for 8 weeks. Conversely, the MOD, HZXYF, LZXYF, and ATO groups were subjected to a HFD (1.25% cholesterol and 20% saturated fat) to induce the desired model. Throughout the study period, ZXYF was administered at doses of LZXYF (3.25 g/kg) and HZXYF (6.50 g/kg) via oral gavage, along with an oral administration of 1.5 mg/kg atorvastatin (ATO). These dosages were based on our previous research findings [10]. The experimental design of this study is illustrated in Fig. 1.

### Cell experiment

Cell culture: HepG2 cells (CL0103, Procell) and Human colonic adenocarcinoma cells (Caco-2) cells were maintained in high-glucose DMEM medium supplemented with 10% or 20% fetal bovine serum (FBS) and 1% penicillin/streptomycin (v/v). Cells were incubated at 37 °C in a humidified atmosphere containing 5% CO₂. Medium was refreshed every 48–72 h. Cells were subcultured at 80–90% confluency using 0.25% trypsin–EDTA.

Cell viability: HepG2 cells and Caco-2 cells were carefully seeded in 96-well plates at a density of 1 × 10^4^ cells per well. Subsequently, the cells were treated with varying concentrations of ZXYF, ranging from 256 mg/ml to 1 mg/ml, as well as a control group with 0 μg/ml, for a duration of 24 h. Following this incubation period, the CCK8 commercial kit was employed to assess cell viability, strictly adhering to the manufacturer's protocol, Cell viability = [(experimental hole OD—blank hole OD)/(control hole OD—blank hole OD)] × 100%.

Cell treatment: Caco-2 cells were divided into 6 groups: Control (standard culture medium), FFA [medium containing FFA mixture: 250 μM palmitic acid (PA) + 500 μM octadecenoic acid (OA)], FFA + L/M/H ZXYF (2/4/6 mg/ml), FFA + FXR agnoist (5 μM Fexaramine) + HZXYF (6 mg/ml); HepG2 cells were divided into 4 groups: Control (standard culture medium), FFA [medium containing FFA mixture], FFA + ZXYF (6 mg/ml), FFA + FXR inhibitor (5 μM GSK2033) + HZXYF (6 mg/ml). To establish a lipid accumulation model, free fatty acids were prepared by blending 20% free bovine serum albumin (BSA) with a 2:1 ratio of OA (B21592, Shanghai yuanye) and PA (P346928, Shanghai yuanye). The mixture was dissolved and suspended through ultrasonic treatment. The final concentration of FFA was 750 μM. The cells were counted and seeded into a 6-well plate and treated with FFA or/ and different reagents for 24 h.

Cell sample preparation: Following cell collection, PBS was added to the sample. A cell suspension was prepared using 100ul of PBS for every million cells, ensuring homogeneity. The cells were then subjected to ultrasonic disruption under an ice-water bath to maintain sample integrity. Subsequently, the samples were centrifuged at 3500 rpm for 15 min, and the resultant supernatant was carefully collected for further testing.

Cell staining: HepG2 cells were washed with PBS after removing the culture medium, fixed in 10% neutral formaldehyde for 10 min, and rinsed again with PBS. Following incubation with a blocking solution for 10 min, cells were stained with Oil Red O working solution for 20 min, briefly rinsed with decolorizing solution, and counterstained with hematoxylin for 20 s. Samples were mounted with glycerol gelatin and imaged under an optical microscope (Leica, Germany).

Transepithelial electrical resistance (TEER) measurement: Caco-2 cell monolayers were employed, and changes in intestinal barrier function were determined through TEER. Caco-2 cells were seeded onto Transwell inserts (polycarbonate membrane, 0.4 μm pore size, Corning) at a density of 1 × 10^5^ cells/cm^2^. The culture medium volumes were maintained at 0.5 and 1.5 mL in the apical and basolateral compartments, respectively. The cells were differentiated for 21 days, and the medium was refreshed every 2 days. TEER values were monitored using a Millicell ERS-2 Volt-Ohm Meter (Merck Millipore). The background resistance (*R*_blank_) of cell-free Transwell inserts filled with culture medium was measured. Electrodes were sterilized with 70% ethanol and rinsed with PBS before use. The apical and basolateral compartments of the Transwell system were equilibrated with pre-warmed (37 °C) culture medium for 10 min. TEER values (*R*_sample_) were recorded by placing the electrodes in contact with the apical and basolateral media. Cell monolayers with stable TEER values exceeding 500 Ω were utilized. For the treatment, differentiated Caco2 monolayers were pretreated with ZXYF for 24 h and then exposed to FFA for 24 h. TEER (Ω⋅cm^2^) = (*Rsample*—*R*_sample_) × membrane area(cm^2^).

FITC-dextran: To assess paracellular permeability, 0.5 mL of FITC-dextran (4 kDa; 1 mg/mL in HBSS) was added to the apical chamber, while 1.5 mL of HBSS was added to the basolateral chamber. Plates were protected from light and incubated at 37 °C for 2 h. Subsequently, 100 μL aliquots were collected from the basolateral chamber and diluted 1:1 with HBSS. Fluorescence intensity was measured using a microplate reader (excitation: 490 nm, emission: 520 nm). A standard curve of FITC-dextran (0.1–10 μg/mL) was used to quantify concentrations. Apparent permeability coefficients (Papp) were calculated using the equation:$Papp=\frac{\Delta C\cdot V}{A\cdot C0\cdot \Delta t}$. Where ΔC is the basolateral concentration of FITC-dextran, V is the basolateral volume (mL), A is the membrane surface area (cm^2^), C0 is the initial apical concentration (μg/mL), and Δt is the incubation time (s).

### The detection of lipids and total bile acids

After an 8-week feeding period, all mice were weighed and sedated with a 1% solution of pentobarbital sodium (40 mg/kg, i.p.). Blood samples were obtained by enucleating the eyeball and subsequently centrifuged at a speed of 5000 rpm for 10 min before being stored at − 20 °C. Tissue, fecal samples and cell sample were prepared by adding normal saline to the sample in a tube at a weight ratio of 1:9. The tissue or stool sample was then pulverized in an ice-water bath to form a homogenate. Subsequently, the specimens underwent centrifugation at 3500 rpm for fifteen minutes, after which the supernatant was collected for examination [12]. Fully automatic biochemical analyzer (AU5800, Beckman Coulter, USA) was used along with TC test kit, TG triglyceride test kit, HDL-C test kit, LDL-C test kit (Qiangsheng Biotechnology Co., Ltd, Zhejiang, China), and total bile acids (TBA) Aassay kit Colorimetric (K209-100, BioVision, USA) following manufacturer guidelines.

### Aortic lesion analysis

The aortic lesion analysis was conducted using hematoxylin and eosin (H&E), Oil Red O, and Masson’s trichrome staining, in accordance with previous studies [8]. In brief, after the blood collection, the mice underwent transcranial perfusion with 0.1 mol/L PBS, followed by fixation in 4% paraformaldehyde (PFA). The heart and aorta were extracted, calibrated, and then immersed in an OCT medium. Subsequently, the cardiac tissues were sectioned into consecutive slices (10 μm) using a microtome (Leica, Germany), starting from the aortic sinus to the aortic arch. The subsequent cross-sections of the aortic root were then stained with Oil Red O, H&E, and Masson's trichrome for histological analysis. An automatic positive fluorescence microscope (Zeiss, German) was used to view the staining outcomes of the aortic sinus. The subsequent step involves the utilization of Image Pro Plus 6.0, an image analysis software developed by Media Cybernetics in Rockville, MD, USA. This software was employed to conduct computational analysis. The following equation was utilized to carry out the semi-quantitative analysis. The semi-quantification was performed using the following formula: Area of plaque (PA, mm^2^) = vessel area (IE, mm^2^)-lumen area (LA, mm^2^), Oil Red O-positive staining area (%) = the Oil Red O-stained area/lumen area. The Collagen-positive area of plaque (%) = the area of Masson’s trichrome-stained collagen/lumen area [9].

### Enzyme-linked immunosorbent assay

The supernatants were obtained by centrifuging at 4 °C, 12,000 rpm for 10 min, followed by determination of the total protein content using a BCA commercial kit. The ileal, serum and caco2 cell supernatant concentrations of LPS (KTE71161, Abbkine, China), IL-1β (PI301, Beyotime, China), IL-6 (PI326, Beyotime, China), IL-10 (PI523, Beyotime, China), and TNF-α (PT512, Beyotime, China) were analyzed using an ELISA kit. Throughout all experimental procedures, strict adherence to the instructions provided with the kit was maintained.

### Measurement of intestinal permeability

The intestinal permeability was assessed using the FITC-labeled dextran method, which involves the utilization of fluorescein isothiocyanate, to examine and evaluate intestinal permeability. In conclusion, FITC-dextran (Sigma-Aldrich, USA) with a molecular weight of 4000 was orally administered to mice at a dosage of 600 mg/kg. Subsequently, serum samples were collected for further analysis. The amount of FITC-Dextran 4 (FD4) present in the serum was measured using a fluorescence spectrophotometer configured to an excitation wavelength of 485 nm and an emission wavelength of 535 nm [10].

The ileal samples were preserved in 4% paraformaldehyde for H&E staining, then dehydrated using ethanol in paraffin, and sliced to a thickness of 5 μm. Subsequently, these segments were dyed using H&E.

In the immunohistochemistry study (IHC), the tissue sections were subjected to antigen retrieval by placement in EDTA antigen repair buffer (pH 8.0). Endogenous peroxidase activity was quenched using 3% hydrogen peroxide for a duration of fifteen minutes, followed by rinsing with PBS for ten minutes. Subsequently, the sections were blocked with a solution containing 5% BSA/0.3% TritonX100/PBS for thirty minutes. Sections were incubated with the primary antibodies ZO-1 (21,773–1-AP, dilution: 1:500, Proteintech) or Claudin1 (13,050–1-AP, Proteintech) was diluted 1:500 and left overnight at 4 °C. After being washed three times with PBS, the secondary antibodies were added at room temperature. Subsequently, it was subjected to staining using diaminobenzidine (DAB) for a duration of 50 min, followed by a brief 10-s treatment with a hematoxylin solution and ultimately mounted with neutral balsam. The positive staining area was quantified utilizing image J software after capturing images under a light microscope [11].

For immunofluorescence staining (IF), the ileal sections were prepared according to previously described methods. After a 30-min incubation at room temperature, the ileal sections were washed three times with PBS. Antigen retrieval was performed using a temperature-controlled water bath set at 90 °C for 10 min in a citric acid buffer with a pH of 6.0. Subsequently, the sections were blocked with 5% BSA and 0.2% Triton X-100/PBS for one hour, followed by overnight incubation at room temperature with anti-MUC2 antibody (27,675–1-AP, dilution: 1:200, Proteintech) for one hour. The next day, the slices were treated with Alexa Fluor 488 goat anti-rabbit antibody at room temperature for 60 min [12].

### The process of extracting DNA from microbes and sequencing the 16S rRNA gene

The E.Z.N.A.-soil DNA kit (Omega Bio-Tek, Norcross, GA, USA) was employed to extract microbial DNA from fresh excrement according to the manufacturer’s instructions. Subsequently, DNA purification and concentration were evaluated using the NanoDrop instrument (Thermo Science, USA). Typically, 2–3 stool samples were collected from each mouse group. A fecal DNA extraction kit was utilized for isolating DNA from the feces prior to Illumina Miseq sequencing. Raw sequencing data underwent filtration and processing before analyzing bacterial community composition in terms of quantity and diversity through efficient clustering of operational taxonomic units (OTUs) and species categorization [13]. Differences in species composition among sample groups were determined by comparative cluster analysis Statistical methods based on QIIME and R packages (v3.2.0), following established protocols for 16S rRNA gene sequencing analysis, were applied to analyze bacterial community structure at different levels.

### Untargeted metabolomics study

Following the treatment, a 200 mg sample of defrosted feces and 1 mL of methanol were transferred into a 1.5 mL centrifuge tube. The mixture was prepared and incubated for 20 min at 4 °C prior to utilization. After centrifugation at 12,000 rpm for 10 min at 4 °C, the samples were collected, removing any solid debris. Subsequently, the supernatant was transferred and filtered using a 0.22 μm filter. The UHPLC-MS system was then injected with 4 μL of the supernatant. The Supplementary material (1.2 Untargeted metabolomics study) provides details on analysis conditions and data processing procedures.

### LC–MS-based quantification of specific bile acids

The method for quantitatively analyzing the bile acid phenotype and concentration in the feces of ApoE^−/−^ mice is based on previously published studies. Briefly, the specimens were processed in 400 μL of methanol (− 20 °C) with the addition of 100 mg of glass beads and vigorously mixed for 60 s. The samples were then placed into a tissue pulverizer and crushed at a frequency of 55 Hz for one minute. This procedure was repeated at least two more times. Ultrasound treatment was performed at room temperature for 30 min, followed by centrifugation at 12,000 rpm and 4 degrees Celsius for 10 min. Supernatant (300 μl) was collected and mixed with water (600 μl), then vortexed for 30 s. An appropriate amount of the supernatant was diluted tenfold with 30% methanol. The liquid above the precipitate (referred to as the supernatant) was filtered through a membrane filter with a pore size of 0.22 μm. The filtered solution was transferred to the LC–MS container, followed by measurement and mixing of bile acid standard with methanol to create a final concentration of 1000 μg/mL for the mixed standard stock solution. This stock solution was further diluted using 30% methanol to obtain ten points on the standard curve. Targeted examination of BA utilized an ACQUITY UPLC® BEH C18 column (2.1 × 100 mm, Waters, USA). All working standard solutions and stock solutions were stored at − 20 °C.

### Reverse transcription-quantitative polymerase chain reaction (RT-qPCR)

The total RNA concentration was determined using the Nanodrop 2000 spectrophotometer from Thermo Fisher Scientific, USA. The Prime Script™RT Master Mix, manufactured by TaKaRa Bio Inc., Japan, was employed for reverse transcription. The Q-RT-PCR process utilized the ChamQ Universal SYBR qPCR Master Mix developed by Vazyme Biotech Co., Ltd., China. The primer sequences are provided in Table S2. Gene expression levels were calculated as relative fold changes using the 2∆∆CT method.

### Molecular docking

An automated docking study using the AutoDock Vina 1.1.2 package was conducted to explore potential binding sites of Alisol A/B/C, Atractylenolide I/II/III, and Monotropein as ligands of FXR. The structures of Alisol A/B/C, Atractylenolide I/II/III, and Monotropein were obtained from the PubChem database, while the human FXR (PDB code: 7C92) structure was retrieved from the RCSB Protein Data Bank. The binding site of FXR was determined with the following coordinates: center_x: 14.533, center_y: 23.548, and center_z: 23.548, and dimensions size_x: 64, size_y: 68, and size_z: 84. PyMol 2.6.0 software was utilized for the analysis of ligand interactions, including the identification of amino acid residues involved, types of chemical bonds, and the strength of interactions.

### Western blot (WB) analysis of liver and ileal tissues

The process of extracting total protein from liver and ileal tissues involved lysis in a buffer that contained both protease and phosphatase inhibitors. This was then followed by quantification using the BCA method, as provided by Pierce, based in Rockford, Illinois, USA. Proteins were separated by 8–12% SDS-PAGE electro­phoresis and transferred to polyvinylidene fluoride (PVDF) membranes. Following a 2-h blockage with 5% skim milk in a Tween-Tris buffered saline (TBST) solution at room temperature, the primary antibody was introduced. This included fibroblast growth factor 15 (FGF-15, ab229630, abcam), ATP-binding cassette, sub-family G, member 5 (ABCG5, 27,722–1-AP, proteintech); ATP-binding cassette, sub-family G, member 8 (ABCG5, 24,453–1-AP, proteintech); cytochrome P450 family 7, subfamily a, polypeptide 1 (CYP7A1, orb539102, Biorbyt), family 8, sub­family b, polypeptide 1 (CYP8B1, AB19630, abcam), family 27, sub­family a, polypeptide 1 (CYP27A1, AB126785, abcam), farnesoid X receptor (FXR, A9033A, Invitrogen), and GAPDH (Proteintech, 10,068–1-AP). The mixture was then incubated at 4 °C overnight. During the night at 4 degrees Celsius. Following three TBST washes, the membrane was exposed to the secondary antibody for a duration of 2 h at ambient temperature. Subsequently, ECL observed the blot and it was displayed as a density in comparison to GAPDH. The gray value was quantified using ImageJ software.

### Statistical analysis

The mean values with standard deviations (± SD) were presented as outcomes. Student's t-tests were used to assess differences between two groups, while ANOVA followed by Dunnett's test was employed for comparisons among more than two groups. All bar charts in this study were generated using GraphPad Prism 9.0 (GraphPad Software, San Diego, USA). Comparative analysis was performed using the Mann–Whitney U test or Wilcoxon rank-sum test via SPSS 20.0 (IBM SPSS, USA). Significance levels were set at *p < 0.05, **p < 0.01, and ***p < *0.001*.

## Results

### ZXYF ameliorated hypercholesterolemia and atherosclerosis induced by HFD in ApoE^−/−^ mice

**Fig. 2:**
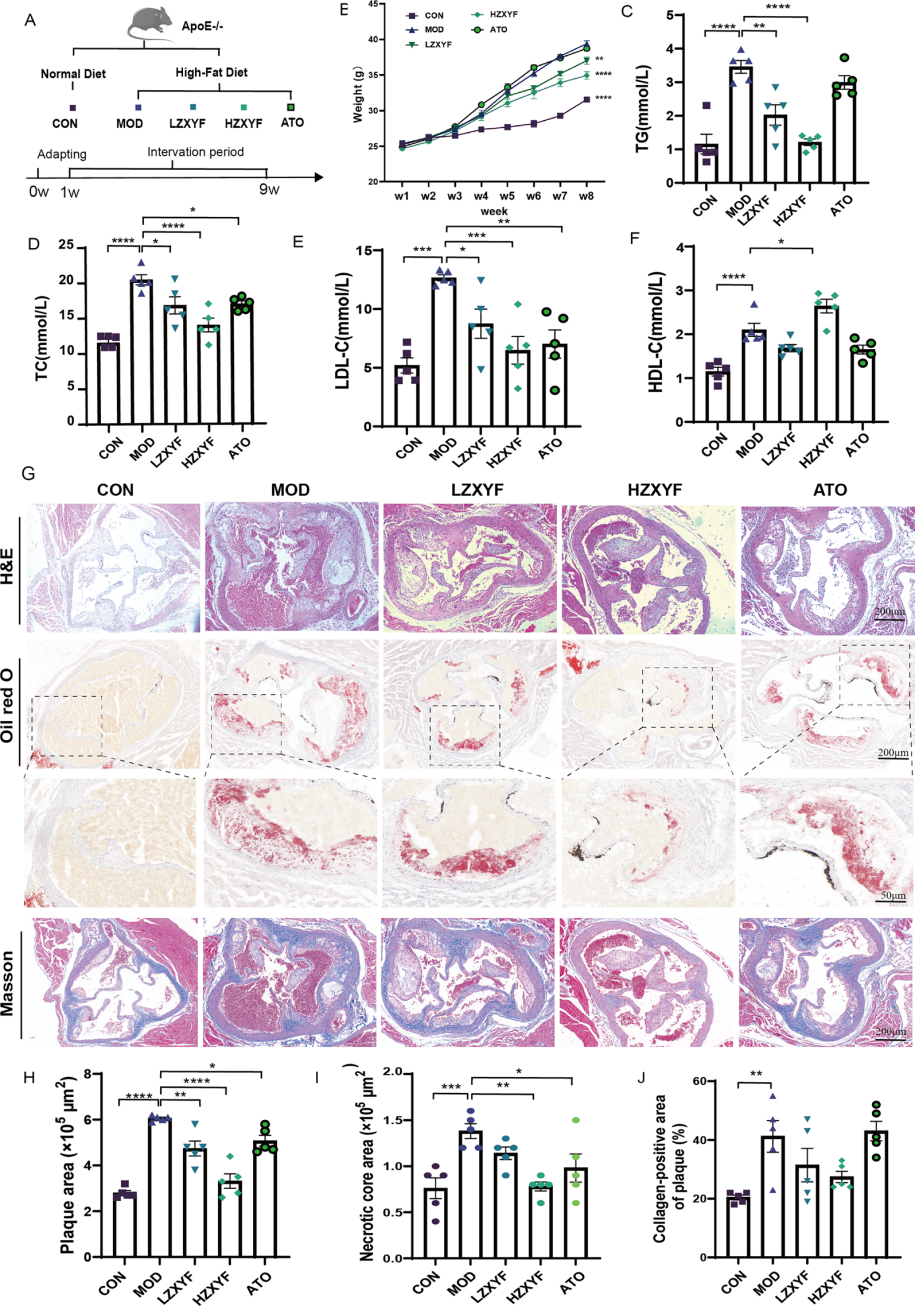
Effect of ZXYF on hypercholesteremia and atherosclerotic plaque in ApoE^−/−^ mice. **A** Schematic of animal experimental design and treatment regimens; **B** Longitudinal monitoring of body weight dynamics during the 8-week intervention period; **C** total cholesterol (TC), **D** triglycerides (TG), **E** low-density lipoprotein cholesterol (LDL-C), and **F** high-density lipoprotein cholesterol (HDL-C); **G** Representative histological sections of aortic roots from ApoE^−/−^ mice: hematoxylin & eosin (H&E) for tissue morphology, Oil Red O for lipid deposition, and Masson's trichrome for collagen visualization (scale bars: 200 μm); **H** Quantitative examination of the percentage of plaque area compared to the vascular lumen area and necrotic core area (**I**); **J** Collagen-positive area of plaque (%). Collagen-Positive Area (%) = (Collagen-Positive Area/ Plaque Area) × 100%; Statistical significance assessed by one-way ANOVA with Tukey's multiple comparisons test (*p < 0.05, **p < 0.01, ***p < *0.001*).

To investigate the defensive effects of ZXYF against dyslipidemia and atherosclerosis, ApoE^−/−^ mice were fed HFD to induce AS and treated with daily doses of ZXYF (Low 3.25 g/kg and 6.50 g/kg) or atorvastatin (10 mg/kg) for 8-week (Fig. 2A). During the intervention, we closely monitored the fluctuations in weight among these groups. Compared to the CON group, the MOD group exhibited a significant increase in body weight induced by HFD, whereas both doses of ZXYF effectively attenuated weight gain (Fig. 2B). After the intervention completed, we assessed serum levels of lipid markers, encompassing TG (Fig. 2C), TC, HDL-C, and LDL-C. We found the MOD group showed a significant increase in serum levels of TC, TG, HDL-C, and LDL-C, while the ZXYF and ATO groups demonstrated a decline in TC and LDL-C levels (Fig. 2D, E). Importantly, HZXYF exhibited a significant reduction in serum TG levels concomitant with an elevation in serum HDL-C levels (Fig. 2C, F). Furthermore, we observed that ZXYF treatment also led to a decrease in the serum concentration of proinflammatory cytokines (Figure S2). In addition, we utilized histological techniques including H&E, oil red O, and Masson staining to assess the development of atherosclerotic lesions in the aortic sinus (Fig. 2G). The H&E and oil red O staining revealed that the MOD group exhibited significantly increased areas of aortic lesions and necrotic plaque regions, whereas treatment with ZXYF and atorvastatin effectively attenuated this impact by markedly reducing the size of aortic plaques (Fig. 2H, I). However, there were no statistically significant differences observed between the MOD and HZXYF groups in terms of aortic fibrosis areas as indicated by Masson staining results (Fig. 2J). Overall, these findings suggest that ZXYF effectively mitigates atherosclerotic lesions induced by a HFD while demonstrating dose-responsive effects on plasma lipid profiles.

### ZXYF treatment enhanced the integrity of the intestinal barrier in HFD induced AS mice

**Fig. 3:**
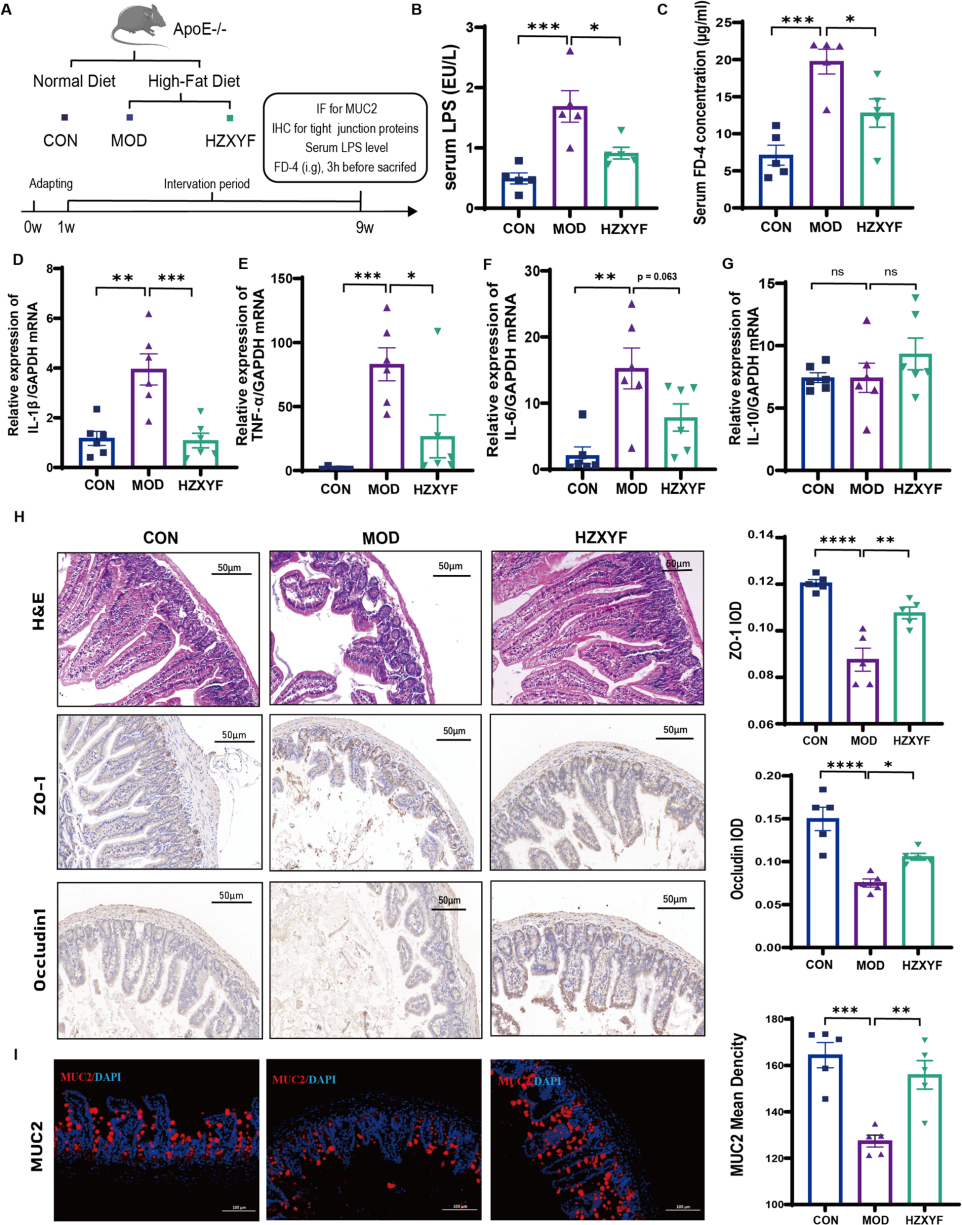
Effect of ZXYF on intestinal barrier function in HFD-induced AS mice. **A** Schematic representation of animal grouping and experimental timeline; **B** Serum lipopolysaccharide (LPS) concentrations across groups; **C** Quantification of intestinal permeability by serum fluorescein isothiocyanate-dextran 4 (FD-4) levels; **D**–**F** Ileal mRNA expression profiles of pro-inflammatory cytokines: (**D**) *Il1b* (IL-1β), (**E**) *Tnf* (TNF-α), (**F**) *Il6* (IL-6), and **G** anti-inflammatory cytokine *Il10* (IL-10) in ApoE^−/−^ mice. **H** Representative immunohistochemical staining of tight junction proteins ZO-1 and Occludin in ileal epithelium (scale bar: 50 μm); **I** Mucin-2 (MUC2) protein distribution visualized by immunofluorescence (scale bar: 100 μm); Data presented as mean ± SEM (n = 5-6 per group). Statistical significance determined by one-way ANOVA with Tukey’s post hoc test (*p < 0.05, **p < 0.01, ***p < *0.001*)

The disruption of the intestinal barrier is widely acknowledged as a pivotal factor contributing to the pathogenesis of AS [14]. Therefore, we further analyze the intestinal barrier function in HFD-induced AS mice (Fig. 3A). Lipopolysaccharide (LPS) is continuously synthesized by gut bacteria in the intestinal lumen. The efficacy of the intestinal barrier can be assessed by monitoring serum LPS levels, as a robust intestinal barrier effectively impedes LPS infiltration into the bloodstream (Zhao et al., 2024). In this study, a significant increase in serum LPS levels induced by the HFD was observed, which was effectively attenuated upon treatment with HZXYF (Fig. 3B). Additionally, we assessed intestinal permeability through the oral administration of FITC-labelled dextran and subsequently collected serum samples 3 h post-administration for further analysis. In line with earlier findings, the HZXYF group showed a reduction in the circulating concentration of DX-4000-FITC (Fig. 3C). Simultaneously, HZXYF effectively reduced the levels of intestinal inflammation factors IL-1β (Fig. 3D), and TNF-α (Fig. 3E), while demonstrating no impact on the level of and IL-6 (Fig. 3F) and IL-10 (Fig. 3G). These findings suggest that HZXYF has a beneficial effect in mitigating HFD-induced intestinal inflammation. Furthermore, the histological analysis using H&E staining revealed that the intestinal mucosa in the CON group remained intact and preserved its original morphology, exhibiting well-aligned intestinal glandular structures and an unaltered lamina propria. In contrast, the MOD group displayed significant pathological alterations including mucosal and submucosal layer swelling, damaged glands, villi loss, and crypt disappearance (Fig. 3H). The HZXYF treatment partially protects against the injuries induced by HFD, resulting in a higher preservation of the small intestinal mucosal epithelium, relatively well-organized glands, and inhibition of mucosal layer necrosis. We conducted a thorough investigation into the expression of key intestinal tight junction proteins (TJs), ZO-1 and Claudin1, which are involved in the connection of adjacent epithelial cells. IHC analysis revealed a significant decrease in ZO-1 (Fig. 3) and Claudin1 (Fig. 3I) expression in the ileum of the MOD group compared to the CON group, while treatment with HZXYF notably upregulated their expression levels. Furthermore, IF was performed to evaluate the secretion of MUC2 expression by mucus goblet cells. Administration of HZXYF also led to an elevation in the protein expression of MUC2 (Fig. 3H, K). These findings indicate that HZXYF treatment alleviated intestinal inflammation and enhanced intestinal barrier functions in the AS mice.

### ZXYF treatment mitigates HFD induced gut dysbiosis in ApoE^−/−^ mice

**Fig. 4:**
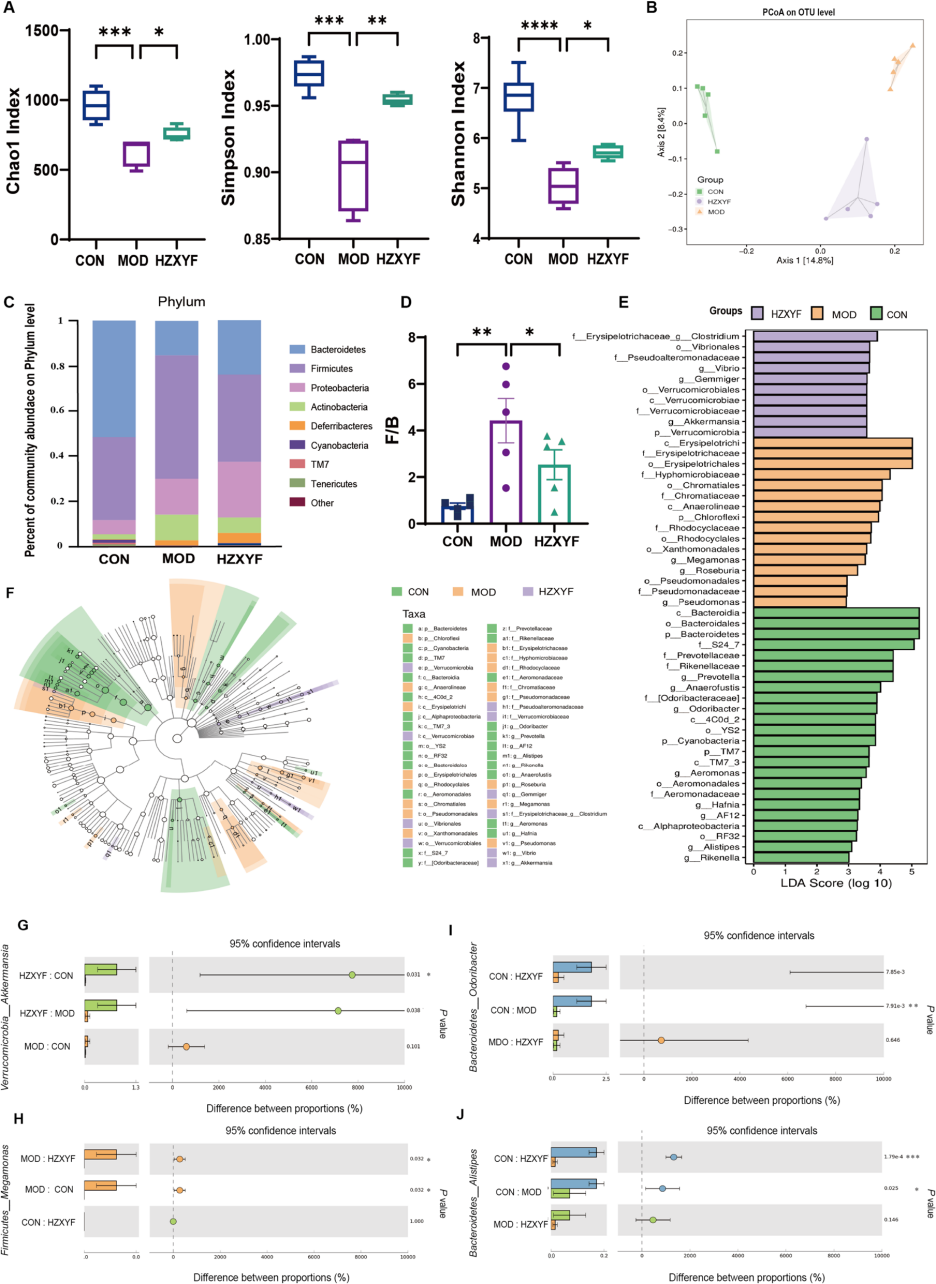
ZXYF treatment remodels the gut microbiota in AS mice. **A** Alpha diversity indices (Chao1 richness, Simpson diversity, and Shannon diversity) calculated for each group; **B** Principal coordinates analysis (PCoA) of beta diversity based on Jaccard distance matrix; **C** Taxonomic composition of gut microbiota at the phylum level, presented as relative abundance (%) of total sequencing reads; **D** Firmicutes/Bacteroidetes (F/B) ratio comparisons among groups, with horizontal lines indicating group medians; **E** Linear discriminant analysis (LDA) effect size (LEfSe) results identifying differentially abundant microbial features; **F** Phylogenetic cladogram of LEfSe-identified taxa with significant inter-group differences. **G**–**J** Inter-group comparisons of relative abundances of *Akkermansia* (**G**), *Megamonas* (**H**), *Odoribacter* (**I**), and *Alistipes* (**J**). LDA score > 3.0, *p* < 0.05 by Kruskal–Wallis test. one-way ANOVA with Tukey's post hoc test. Data expressed as mean ± SEM (n = 5 per group, *p* < 0.05, *p < 0.01, **p < 0.001)

Considering the significance of intestinal microbiota and its metabolites in the development of AS and intestinal barrier function, we assessed structural changes in microbiota in response to ZXYF administration. The overall composition of the intestinal microflora in the HZXYF group exhibited slight differences compared to that of the MOD group. In this study, the gut microbiota in the MOD group demonstrated reduced richness and diversity compared to the CON group, as indicated by the Chao1, Simpson, and Shannon indices. However, HZXYF significantly ameliorated these differences (Fig. 4A). Principal coordinate analysis (PCoA) based on Jaccard matrices revealed a clear clustering pattern in the microbiota composition among the CON, MOD and HZXYF groups (Fig. 4B). An analysis at the phylum level revealed that *Firmicutes*, *Bacteroidetes*, and *Proteobacteria* were the predominant subgroups observed in the alterations of gut microbiota composition (Fig. 4C). Notably, the average *Firmicutes/Bacteroidetes* (F/B) ratio in the MOD group was significantly increased compared to the CON group, and this increase was partially restored after HZXYF treatment (Fig. 4D). The specific changes in genera within the MOD and HZXYF groups were further compared. In addition, gut microbial groups were identified with statistical significance using an LDA score of 2.0. The online analysis of LDA Effect Size (LEfSe) revealed a higher prevalence of *Prevotella* and *Anaerofustis* in the CON group, while the MOD group exhibited a greater concentration of *Megamonas* and *Roseburia*. Following HZXYF treatment, dominant strains included *Vibrio*, *Gemmiger* and *Akkermansia (AKK)* (Fig. 4E, F). Furthermore, we investigated the abundance levels of *Akkermansia*, *Megamonas*, *Odoribacter*, and *Alistipes* among these three groups. Results show a significant enrichmend of *AKK* in HZXYF group (Fig. 4G–J). Above all, we found asubstantial alteration in gut microbial diversity after ZXYF treatment.

### ZXYF treatment modified macrobiotics metabolomic phenotypes in AS mice

**Fig. 5:**
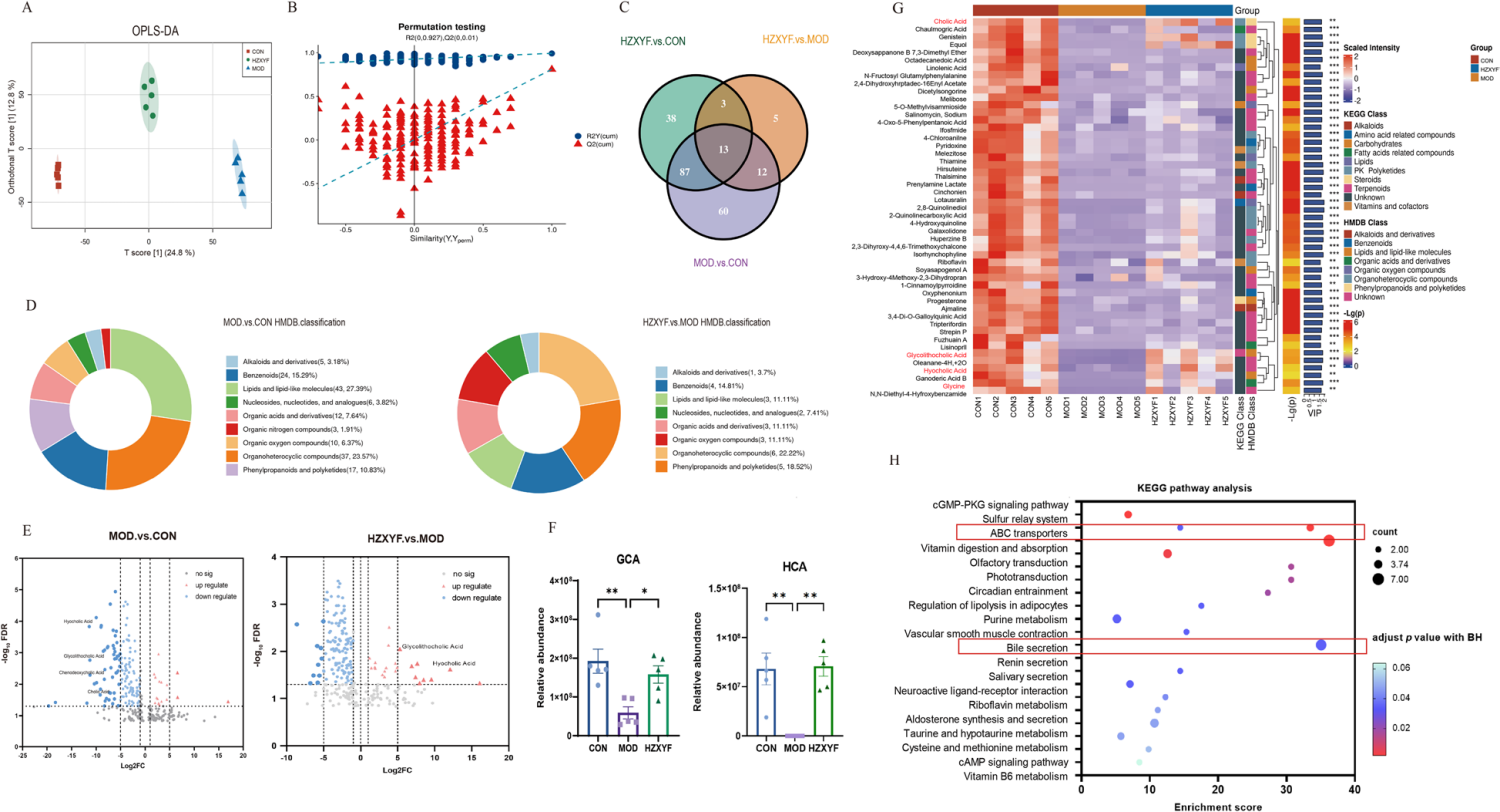
ZXYF treatment reshaped microbiotic metabolomic phenotypes in ApoE^−/−^ mice. **A** Orthogonal partial least squares-discriminant analysis (OPLS-DA) score plot demonstrating metabolic separation among three experimental groups after 8-week intervention; **B** Permutation test validation of OPLS-DA model robustness; **C** The Venn diagram of differential metabolites in three groups; **D** Chemical classification of differential metabolites according to Human Metabolome Database (HMDB) superclasses; **E** Volcano plots comparing metabolite alterations between MOD vs. HZXYF (left) and HZXYF vs. MOD (right) groups; vertical dashed lines denote log2(1/1.5) and log2(1.5); **F** Group-wise comparisons of primary bile acids: glycocholic acid (GCA) and hyocholic acid (HCA); **G** Hierarchically clustered heatmap of top 50 differential metabolites (Z-score normalized); **H** Pathway impact analysis via KEGG enrichment, with DAScore quantifying pathway perturbation significance; Data expressed as mean ± SEM (*n* = 4–5 per group). Statistical significance determined by one-way ANOVA with Benjamini–Hochberg correction (*p* < 0.05, *p < 0.01)

An untargeted metabolomics analysis was performed to further assess the changes in metabolites induced by HZXYF. The metabolic profiles of fecal samples from ApoE^−/−^ mice were determined using UHPLC-Q-Exactive LC–MS, with both negative and positive electrospray ionization (ESI) modes utilized. The Orthogonal Partial Least Squares Discriminant Analysis (OPLS-DA) and its Permutation test revealed a clear separation pattern among the CON, MOD, and HZXYF groups (Fig. 5A, B). In total, there are 218 statistically significant metabolites were identified in the fecal (Fig. 5C). By leveraging the HMDB database, we conducted a comprehensive classification of the distinct metabolites identified in both the MOD vs. CON group and the HZXYF vs. MOD group. Our findings suggest that these metabolites predominantly consist of lipids and lipid-like compounds, with Benzenoids emerging as a prominent subsequent category (Fig. 5D). According to the volcano maps, a significant decrease was observed among the altered metabolites of the MOD vs. CON group, specifically in four bile acid metabolites: CA, CDCA, hyocholic acid (HCA), and glycolithocholic acid (GCA). However, administration of HZXYF effectively restored the levels of HCA and GCA (Fig. 5E, F). The hierarchical clustering heatmap presented in Fig. 5G illustrates the top 50 metabolites that exhibit significant differences in abundance among the three groups (VIP > 1, *p*-value < 0.05). Furthermore, KEGG pathway enrichment analysis of these metabolites highlighted major pathways such as ABC transpoter and bile secretion (Fig. 5H). The results presented herein demonstrate the robust modulation of cholesterol excretion and bile acids metabolism pathway by ZXYF.

### ZXYF treatment enhances cholesterol excretion and alters fecal BAs profile in AS mice

**Fig. 6:**
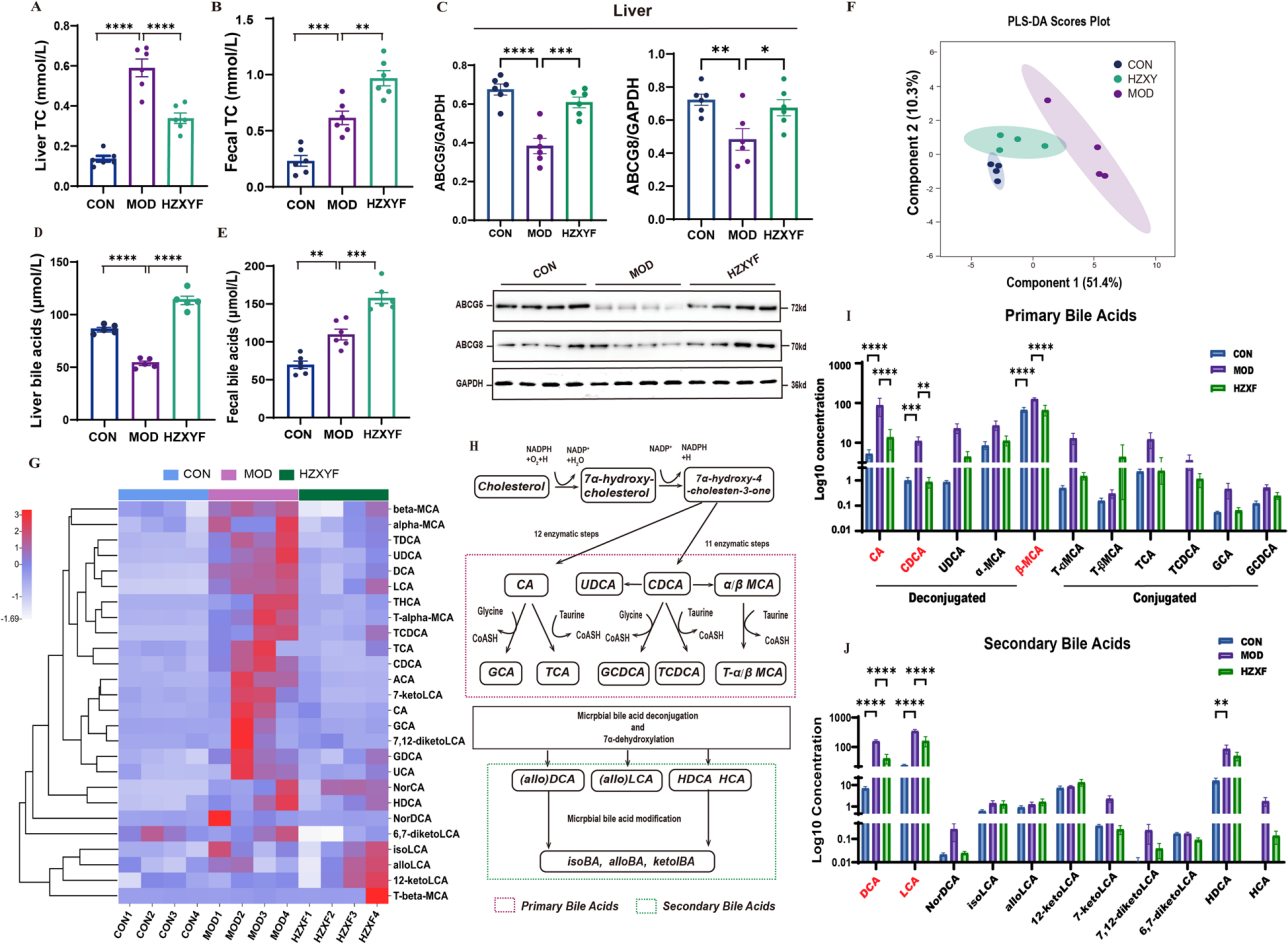
Effect of ZXYF treatment on fecal cholesterol extraction and fecal BAs composition. **A** Total cholesterol (TC) levels in liver tissue; **B** Neutral sterol excretion quantified by fecal TC content; **C** Western blot analysis of hepatic ATP-binding cassette transporters ABCG5 and ABCG8 (Lower: representative immunoblots; Upper: densitometric quantification normalized to GAPDH); **D** Total BA pool size in liver tissue; **E** Fecal BA excretion levels; **F** Partial least squares-discriminant analysis (PLS-DA) of BA compositional profiles; **G** Hierarchical clustering heatmap and relative abundance composition of differentially regulated BAs detected by LC–MS/MS (n = 4 per group); **H** Schematic illustration of BA enterohepatic circulation: primary BAs (PBAs) → secondary BAs (SBAs) transformation via gut microbial metabolism. **I** Ileal concentrations of deconjugated vs. conjugated PBAs; **J** SBAs levels in ileal content; Data expressed as mean ± SEM. Statistical significance determined by one-way ANOVA with Tukey's post hoc test (*p < 0.05, **p < 0.01, ***p < *0.001*)

Based on the untargeted metabolomic results, we further investigated hepatic cholesterol levels, and the key protein involved in cholesterol excretion. In this study, a significant increase in both hepatic and fecal TC levels was observed in the MOD group compared to the CON group. Treatment with HZXYF resulted in a reduction of hepatic TC levels (Fig. 6A) and a further elevation of fecal TC levels in HFD-fed ApoE^−/−^ mice when compared to the MOD group (Fig. 6B). In line with this, a significant increase in liver ABCG5 and ABCG8 expression were observed following HZXYF intervention (Fig. 6C). The metabolism of cholesterol exhibits a significant correlation with the metabolism of bile acids. Compared to the MOD group, a decrease in liver total bile acids (TBA) levels (Fig. 6D) and an increase in fecal TBA levels were observed in the HZXYF groups (Fig. 6E). Furthermore, the targeted metabolomic analysis of bile acids using UPLC-GC/MS revealed that HZXYF induced alterations in fecal bile acid composition, as demonstrated by the PLS-DA (Fig. 6F). The administration of HZXYF partially reversed the alterations in fecal bile acids induced by the HFD, as indicated in the heatmap, showing a closer alignment with those of control mice (Fig. 6G). Conjugated bile acids are formed by the combination of free bile acids with glycine or taurine. Through bacterial activity in the intestine, they undergo hydrolysis and dihydroxylation during bile acid metabolism, leading to their conversion into SBAs (Fig. 6 H). Compared with the MOD group, the administration of ZXYF significantly decreased the levels of fecal primary bile acids (PBAs), including CA, CDCA and β-MCA **(**Fig. 6I**)**. Concurrently, there was a remarkable reducation in the levels of SBAs, including DCA and LCA (Fig. 6J). The collective findings suggest that ZXYF effectively mitigated atherosclerosis induced by a HFD through mechanisms involving reduced hepatic cholesterol levels, enhanced fecal excretion of cholesterol and bile acids, as well as alterations in the composition of fecal bile acids.

### ZXYF-mediated enrichment of AKK and modulation of SBAs levels alleviate HFD-induced intestinal barrier dysfunction and atherosclerosis

**Fig. 7:**
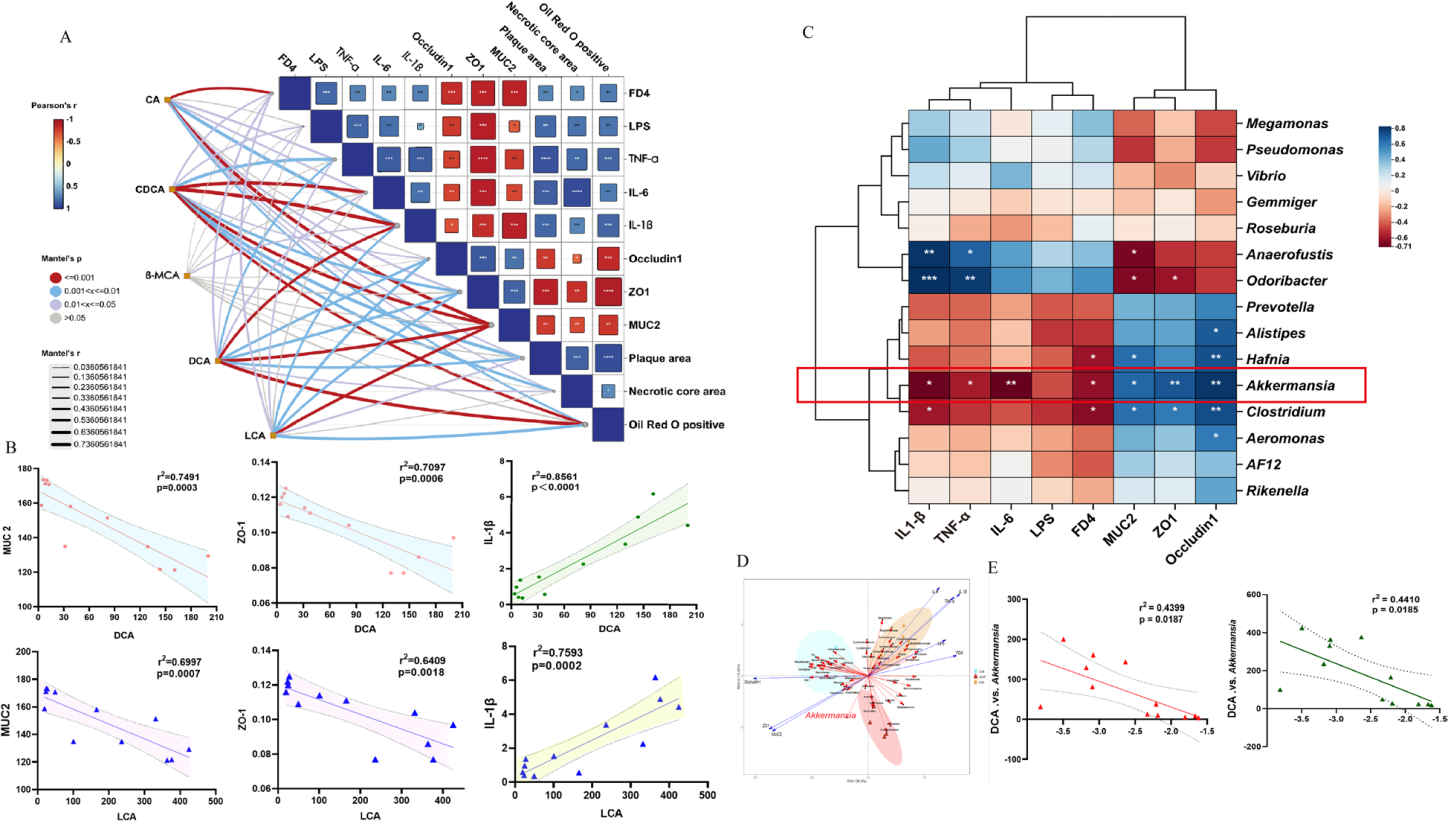
ZXYF-mediated enrichment of AKK and modulation of SBAs levels alleviate HFD-induced intestinal barrier dysfunction and atherosclerosis. **A** Mantel test correlation matrix between major BAs [cholic acid (CA), chenodeoxycholic acid (CDCA), β-muricholic acid (βMCA), deoxycholic acid (DCA), lithocholic acid (LCA)] and key pathophysiological indices: intestinal inflammation (lipopolysaccharide [LPS], TNF-α, IL-6, IL-1β), barrier integrity (FD-4 permeability, ZO-1, Occludin, MUC2), and atherosclerotic lesions (plaque area, necrotic core, Oil Red O^+^area). Heatmap intensity reflects Mantel's *r* with 999 permutations (*p* < 0.05 by Benjamini–Hochberg correction); **B** Pearson correlation network between secondary BAs (DCA, LCA) and intestinal barrier proteins (ZO-1, Occludin, MUC2); **C** Genus-level gut microbiota correlation heatmap with barrier integrity markers (color scale: Z-score transformed *r* values, *p* < 0.05 by two-tailed test); **D** The Redundancy analysis (RDA) triplot unveiled the association between distinct gut microbiota and intestinal inflammation markers (FD, LPS, TNF-α, IL-6, IL1-β) as well as intestinal barrier permeability indicators (FD4, ZO-1, Occludin1, MUC2); **E** Dose-dependent relationship between *Akkermansia* relative abundance and DCA levels; Data expressed as mean ± SEM (n ≥ 4). Statistical significance determined by: (**A**, **D**) Permutation tests; (**B**, **C**, **E**) Pearson correlation with FDR correction

The gut microbiota produces SBAs through a process involving the synthesis of PBAs from cholesterol in liver cells, which then enter the intestine with bile. Intestinal bacterial bile salt hydrolase (BSH) and 7α-hydroxylase mediate the conversion of PBAs into two main types of SBAs, DCA and LCA [15]. Previous studies have demonstrated that HFD induces an elevation in DCA content within the intestines, consequently leading to a reduction in TJs between intestinal epithelial cells, resulting in an increase in intestinal mucosal permeability and compromises the integrity of the intestinal barrier. In our study, we investigated the correlation between intestinal barrier function indices and differential bile acids. The interactive Mantel test correlation heatmap reveals a robust association between IL-1β levels and CDCA and DCA, while the MUC2 level demonstrates a significant correlation with CA, CDCA, DCA, and LCA (Fig. 7A). The subsequent Pearson’s correlation analysis revealed a negative association between secondary bile acids DCA and LCA with intestinal barrier function, while demonstrating a positive correlation with the level of intestinal inflammation (Fig. 7B). The intergroup correlation analysis heatmap further supports that an increase in the abundance of AKK within the Gut microbiota confers beneficial effects on intestinal barrier function (Fig. 7C). Additionally, RDA analysis was performed to explore the association between the differential abundance of microbiota at the genus level regulated by HZXYF and HFD-induced atherosclerosis as well as intestinal barrier dysfunction. The results indicated a more pronounced association between the increased AKK abundance in the HZXYF group and improved intestinal barrier function (Fig. 7D). Further investigation demonstrated an inverse relationship between AKK abundance and levels of bile acids LCA and DCA stimulation (Fig. 7E). Based on these results, it can be speculated that ZXYF may impede the conversion of PBAs into SBAs by increasing AKK abundance, thereby reducing SBAs levels in the distal ileum and ameliorating intestinal barrier dysfunction induced by HFD.

### ZXYF promotes hepatic synthesis of BAs and inhibits BAs reabsorption by activating FXR-FGF15 in AS mice

**Fig. 8:**
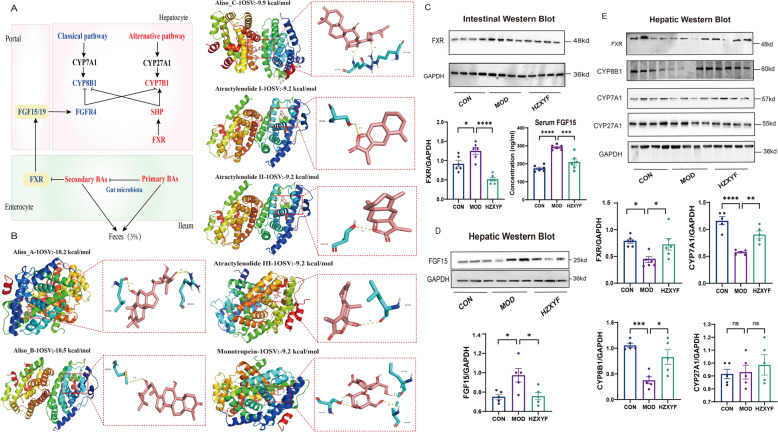
ZXYF treatment regulated BAs enterohepatic circulation and BAs biosynthesis in AS mice through FXR-FGF15 signal pathway. **A** Schematic diagram of BA biosynthesis (classic/alternative pathways) and enterohepatic circulation; **B** Molecular docking analysis of ZXYF active constituents with FXR ligand-binding domain (LBD), with lowest binding energy (-10.5 kcal/mol) shown as representative; **C** Immunoblotting of ileal FXR protein expression; Lower: Serum fibroblast growth factor 15 (FGF15) levels quantified by ELISA; **D** Representative western b lot images of FGF15 and the relative protein expressions of FGF15 in the ileum in ileum of mice; **E** Representative western blot images and relative protein expressions of FXR, CYP8B1, CYP7A1, CYP27A1; Data expressed as mean ± SEM. Statistical significance determined by one-way ANOVA with Tukey's post hoc test (*p < 0.05, **p < 0.01, ***p < *0.001*)

Bile acids are predominantly synthesized by the liver through two distinct pathways: the classic pathway, which is catalyzed byCYP7A1, and the alternative pathway, involving CYP27A1 (Fig. 8A). The liver efficiently reabsorbs 95% of bile acids from the distal ileum through portal blood, highlighting the pivotal role of enterohepatic circulation in bile acid synthesis. The activity of numerous transport proteins is regulated by FXR through the inhibition of bile acid reabsorption at both intestinal and hepatic levels. Moreover, FXR exerts a suppressive effect on bile acid synthesis by inhibiting the expression of CYP7A1. The major components of ZXYF were detected by UHPLC-O-Exactive-Orbitrap/MS (Fig. S1, Table S1). The biding free energy of molecular docking was displayed in Table S3. The results revealed that the binding free energy of Alisol A and Alisol B to the FXR protein crystal (PDBID: 1OSV) was -10.2 and -10.5 kcal/mol, respectively (Fig. 8B). The Western blot analysis revealed a significant upregulation of ileal FXR levels in response to HFD, whereas treatment with HZXYF effectively attenuated this elevation (Fig. 8C). Activation of FXR in the enterohepatic cells releases FGF15, which travels through the portal vein to reach hepatocytes, where it binds with FGFR4 and inhibits CYP7A1, thereby suppressing bile acid synthesis in liver cells. In this study, we observed significantly elevated levels of serum and ileal FGF15 in the MOD group compared to the CON group (Fig. 8C, D). Notably, administration of ZXYF treatment effectively reversed these increases. In the liver, HZXYF treatment resulted in increased expression levels of FXR (Fig. 8E). Moreover, ZXYF treatment led to elevated expression levels of CYP7A1 and CYP8B1, while no significant difference was observed in the expression of CYP27A1 (Fig. 8E). Based on these findings, hepatic bile acid synthesis may contribute to increased de novo BA synthesis partially due to the inhibition of intestinal negative feedback mechanisms by ZXYF.

### The restorative impact of ZXYF on HFD-induced hepatic lipid accumulation and intestinal barrier impairment are dependent on FXR

**Fig. 9:**
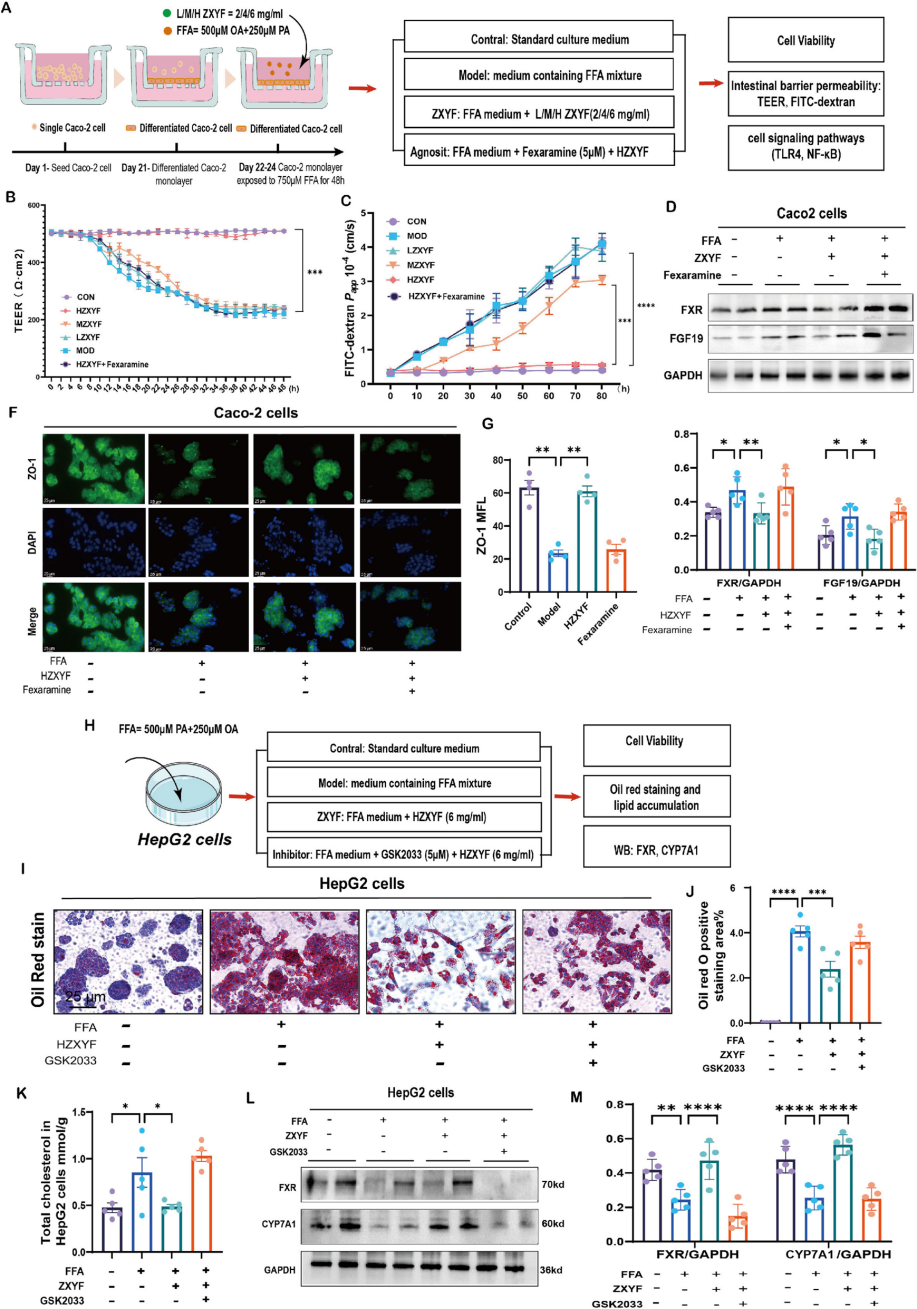
ZXYF ameliorates HFD-induced hepatic lipid accumulation and intestinal barrier dysfunction through FXR-dependent mechanisms. **A** Experimental design for Caco-2 cell interventions modeling intestinal epithelium; **B** Values of differentiated Caco-2 monolayer cells. TEER (Ω⋅cm^2^) = (*R*_*sample*_*—R*_*sample*_) × membrane area(cm^2^); **C** Experimental design for Caco-2 cell interventions modeling intestinal epithelium; **D** Representative immunoblots of FXR and FGF19 in Caco-2 lysates; **E** Relative protein expressions of FXR and FGF19 in Caco-2 cells normalized to GAPDH; **F** Immunofluorescence of ZO-1 protein in Caco-2 cells (scale bar: 25 μm); **G** ZO-1 fluorescence intensity quantification; **H** Experimental scheme for HepG2 hepatocyte lipid loading model; **I** Oil Red O-stained intracellular lipid droplets (scale bar: 25 μm); **J** Quantification of Oil Red O staining area% of HepG2 cells; **K** Total cholesterol (TC) content measured by enzymatic assay (mmol/g); **L** Representative western blot images FXR and CYP27A1 in HepG2 cells; **M** Relative protein expressions of FXR and CYP27A1 in HepG2 cells normalized to GAPDH; Data are expressed as mean ± SEM. (n = 4–5 per group). One-way ANOVA was used to analyse statistical differences; ^*^P < 0.05, ^**^P < 0.01, ^***^P < 0.001

To elucidate the FXR receptor-dependent mechanisms underlying ZXYF’s therapeutic effects on intestinal barrier preservation and lipid metabolism regulation, we conducted pharmacological interventions using Fexaramine (a selective FXR agonist) and GSK2033 (a selective FXR antagonist) in FFA-challenged Caco-2 and HepG2 cell models (Fig. 9 A, H). Prior to experimental interventions, cytotoxicity profiling using Cell Counting Kit-8 (CCK-8) assays established the safety margin of ZXYF in both cell lines (Figure S3 A, B), informing optimal dosing parameters for subsequent investigations.

In polarized Caco-2 monolayers established on Transwell® inserts, ZXYF effectively counteracted FFA-induced barrier dysfunction, demonstrated by significant restoration of transepithelial electrical resistance (TEER) and reduced paracellular permeability to FITC-dextran (Papp) (Fig. 9B, C). Concurrently, ZXYF attenuated FFA-triggered inflammatory responses, as evidenced by decreased TNF-α and IL-6 secretion (Figure S3C, D). Notably, the barrier-protective and anti-inflammatory effects were completely abolished by Fexaramine cotreatment, confirming FXR pathway dependency. Mechanistic investigations through immunoblotting revealed that FFA treatment upregulated FXR and its downstream effector FGF19 while suppressing tight junction protein ZO-1 expression in intestinal epithelium. ZXYF administration normalized these molecular alterations, but FXR hyperactivation via Fexaramine cotreatment nullified the therapeutic effects (Fig. 9D). Parallel investigations in HepG2 hepatocytes demonstrated ZXYF’s capacity to reduce FFA-induced lipid accretion, with Oil Red O staining showing significant decrease in intracellular lipid droplets (Fig. 9 I, J) and corresponding reduction in TC (Fig. 9K). These metabolic improvements were reversed by FXR inhibition via GSK2033. Mechanistically, FXR blockade disrupted bile acid homeostasis, as evidenced by altered expression of rate-limiting enzyme CYP27A1 (Fig. 9L, M). The aforementioned results further demonstrate that the ameliorative effects of ZXYF on intestinal barrier function and lipid metabolism disorders in AS mice are dependent on FXR activation.

## Discussion

Atherosclerosis, a chronic vascular pathology driven by dyslipidemia and endothelial dysfunction, originates from aberrant lipid metabolism that facilitates subendothelial retention of cholesterol-rich lipoproteins. The pathophysiological cascade is amplified through oxidized low-density lipoprotein (LDL)-mediated inflammatory activation, which synergizes with impaired cholesterol efflux mechanisms to promote foam cell formation and plaque progression. Crucially, maladaptive vascular remodeling—characterized by intimal hyperplasia and elastin fragmentation—is directly potentiated by lipid-driven oxidative stress and macrophage polarization. These interconnected events, rooted in systemic lipid homeostasis disruption, collectively establish a self-perpetuating cycle of arterial stiffening, luminal narrowing, and hemodynamic dysfunction, ultimately manifesting as atherosclerotic cardiovascular disease (ASCVD). Therefore, an effective strategy for preventing atherosclerosis is to target cholesterol metabolism [16]. However, due to the intricate and varied pathological mechanisms of AS, statin therapy has demonstrated certain limitations. There is a need for improved agents with higher efficacy and fewer adverse effects. The anti-atherosclerosis of ZXYF have been confirmed in our previous research [13]. For instance, our previous research discovered that ZXYF can enhance plaque stability and prevent plaque rupture and detachment by hindering the conversion of macrophages to the M1 type and averting the activation of NLRP3 inflammasome. Additionally, ZXYF can also ameliorate AS-related cognitive dysfunction by regulating the MAPK pathway in the hippocampus to reverse synaptic proteins and β-amyloid. However, previous studies on the mechanism of ZXYF in the treatment of AS and its complications have mainly focused on inflammatory pathways, and the preparation for its regulation of lipid metabolism has not been further explored. In our current research, we established the AS model by subjecting APOE^−/−^ mice to a HFD for a duration of 8 weeks. Since atorvastatin primarily targets cholesterol metabolism by competitively inhibiting HMG-CoA reductase, the rate-limiting enzyme in cholesterol biosynthesis. This mechanism robustly reduces LDL-C and TC but has limited efficacy on TG. We found atorvastatin show little effect on the HFD induced TG increasing (Fig. 1C), while ZXYF significantly decreased the serum levels of TG, TC, and LDL-C (Fig. 2C, D). Simultaneously, the results obtained from oil red and HE staining demonstrated that ZXYF effectively mitigated the development of aortic plaques and necrotic lipid cores induced by the HFD in APOE^−/−^ mice (Fig. 2G, I). However, the mechanism by which it achieves these outcomes has remained unclear.

Accumulating evidence positions the restoration of HFD-compromised intestinal barrier integrity as a novel therapeutic paradigm for AS pathogenesis [17]. Functioning as a tripartite defense system, the intestinal barrier integrates three synergistic components: (1) the polarized epithelial monolayer with tight junction complexes; (2) gut-associated lymphoid tissue orchestrating immune surveillance; and (3) commensal microbiota maintaining metabolic homeostasis. This multilayered biological interface exhibits dynamic selective permeability, actively preventing microbial translocation while suppressing metabolic endotoxemia through coordinated muco-immunological regulation. Notably, its dysfunction potentiates systemic inflammation via "leaky gut"-mediated pathogen-associated molecular pattern (PAMP) dissemination-a critical mechanistic link bridging intestinal hyperpermeability to atherosclerotic plaque vulnerability. In our study, the administration of HFD was found to be associated with a significant increase in intestinal inflammation and dysfunction of the intestinal barrier, which exhibited notable improvement subsequent to treatment with ZXYF. Specifically, TJs located between epithelial cells, play a crucial role in regulating paracellular permeability within the intestines and maintaining the integrity of the intestinal barrier [18]. Our research findings indicate a significant decrease in ileal concentrations of both transmembrane proteins, specifically occludin-1, and intracellular proteins, namely ZO-1, in AS mice. At the same time, there was a notable increase in serum levels of LPS, an indicative marker of intestinal barrier damage, within the MOD group. Furthermore, a thin layer of mucin layer with increased permeability allows endotoxins to enter the circulatory system, which further activate the inflammatory pathway. Sentinel goblet cells secrete MUC2 to form the mucin layer, which was also reduced in the MOD group. Our research has demonstrated that ZXYF exerts a protective effect on the integrity of the intestinal barrier, as evidenced by enhanced tissue morphology and upregulated expression of occludin1, ZO-1, and MUC2 (Fig. 3H, I). Additionally, there was a concomitant reduction in serum LPS levels and ileal IL-1β and TNF-α levels (Fig. 3B–F). In conclusion, ZXYF has shown significant protective effects on the intestinal barrier by increasing the expression of TJs and reducing inflammation in the ileum.

The symbiotic gut microbiota functions as a central biosensor and metabolic gatekeeper of intestinal barrier homeostasis, orchestrating mucosal defense [19]. We further explore the relationship between alterations in the gut microbiota and intestinal barrier integrity in AS mice following ZXYF intervention. In this study, we observed that ZXYF treatment led to a partial reduction in the F/B ratio (Fig. 4D). Recent research has demonstrated that the F/B ratio serves as a reliable indicator of gut microbiota composition and is closely associated with intestinal barrier function. An elevated F/B ratio signifies an increase in intestinal barrier permeability. However, it is important to note that while the F/B ratio holds significance, maintaining overall balance of gut microbiota is equally imperative [20]. In this study, we have shown that the administration of ZXYF led to an increase in the diversity of gut microbiota, as evidenced by significant changes in the Chao1, Simpson, and Shannon indices (Fig. 4A). Furthermore, we utilized LEfSe to analyze the microbial community data from the CON, MOD group and ZXYF group, revealing a significant increase in AKK genera following ZXYF treatment (Fig. 4E). Clinical studies show significantly reduced levels of AKK in atherosclerotic patients, correlating with disease severity [21]. As a next-generation probiotic, AKK strengthens the intestinal mucus layer by promoting mucin production and tight junction proteins (e.g., occludin, ZO-1). Its supplementation (10^1^⁰ CFU/day) reduces gut inflammation by suppressing pro-inflammatory cytokines (e.g., IL-6) and inhibiting harmful bacteria growth, while enhancing beneficial microbes. These actions improve gut barrier function and may help mitigate atherosclerosis progression through anti-inflammatory and metabolic regulatory effects. Our results have verified that the enhancement of the intestinal barrier and mitigation of atherosclerosis by ZXYF can be attributed, at least in part, to the augmented abundance of AKK (Fig. 7C).

The bidirectional crosstalk between bile acids (BAs) and gut microbiota constitutes a pivotal axis regulating intestinal barrier competence, wherein microbial biotransformation of primary BAs into secondary BAs synergistically activates both host FXR signaling to fortify barrier function. Metabolites produced by the gut microbiota play a pivotal role in mediating communication between the host and the microbiota. Here, Non-targeted metabolomics analysis revealed that all samples included in OPLS-DA indicating a robust separation effect between the CON, MOD, and HZXYF groups (Fig. 5A, B). We quantified the fold change in metabolite abundance and analyzed *P-*values to assess the magnitude of metabolite alterations and changes in associated metabolites. We then performed annotation of differential metabolites in the KEGG database to uncover their biological functions. Metabolomic analyses revealed significant perturbations in both the ABCG5/8 regulatory axis and bile acid enterohepatic cycling (Fig. 5H). The ABCG5/ABCG8 heterodimer, functioning as an ATP-binding cassette transporter, serves as a critical regulatory node in atherogenesis by mediating canalicular cholesterol efflux—a rate-limiting step in biliary cholesterol excretion. This coordinated process reduces plasma cholesterol bioavailability through two synchronized mechanisms: (1) direct elimination of hepatocyte-derived free cholesterol into bile (accounting for ~ 70% of daily cholesterol disposal), and (2) blockade of intestinal cholesterol absorption via apical enterocyte efflux. Dysregulation of this transporter system promotes arterial cholesterol retention, establishing ABCG5/8 as principal molecular determinants of reverse cholesterol transport and atherosclerotic plaque vulnerability [17]. Our study found HFD led to a significant decrease in the levels of ABCG5/8 in the liver of ApoE^−/−^ mice, and this change was reversed by HZXYF treatment (Fig. 6C). The results of gut microbiota metabolomics provided additional evidence for the role of ZXYF in regulating cholesterol metabolism and suggested its connection to bile acid metabolism.

Bile acids play a pivotal role in the pathogenesis of AS, particularly in lipid metabolism and inflammatory responses. They not only act as byproducts of cholesterol metabolism but also actively participate in the digestion and absorption of lipids and cholesterol within the small intestine. Dysregulation of bile acid metabolism is associated with various pathological conditions, including metabolic syndrome and cardiovascular diseases [5]. Based on the findings of the untargeted metabolomics analysis, ZXYF has an impact on the BA metabolism pathway. Notably, our findings reveal a substantial reduction in the levels of total bile acids in the MOD group mice (Fig. 6D). This decline can be attributed to chronic HFD intervention, which induces hepatic progenitor cell dysfunction via lipotoxicity-induced endoplasmic reticulum (ER) stress and epigenetic modifications, thereby reducing bile acid production. Additionally, prolonged HFD intervention enhances the efficiency of the enterohepatic circulation of bile acids, triggering a feedback inhibition mechanism that leads to downregulation of hepatic bile acid synthesis.PBA biosynthesis exhibits species-specific divergence: humans predominantly synthesize CA and CDCA, while rodents produce a broader spectrum including α/β- MCAs alongside CA. Following hepatic synthesis, PBAs undergo amino acid conjugation via bile acid-CoA:amino acid N-acyltransferase (BAAT), forming glycine- or taurine-bound amphipathic molecules essential for lipid emulsification. Post-biliary secretion into the duodenum, gut microbiota orchestrates PBA biotransformation through sequential deconjugation (mediated by microbial bile salt hydrolases), dehydrogenation, and 7α-dehydroxylation, generating SBAs like DCA and LCA. The enterohepatic circulation recaptures ~ 95% of SBAs via apical sodium-dependent bile acid transporter (ASBT)-mediated ileal reabsorption, while the residual fraction (< 5%) undergoes fecal excretion—a critical pathway for cholesterol elimination and microbial homeostasis maintenance. Here, the quantified analysis of TBA levels indicates a significant decrease in serum and liver TBA after ZXYF administration, while the fecal TBA level showed a remarkable increase (Fig. 6A, B, E) [22]. We further investigated the composition of fecal bile acids using targeted LC–MS. The results indicated that ZXYF treatment led to an increase in levels of PBAs in feces. However, ZXYF did not have a significant effect on the levels of conjugated bile acids in feces (Fig. 6I). Interestingly, compared to the MOD group, ZXYF significantly decreased the levels of SBAs, including LCA and DCA (Fig. 6J). The microbial-derived secondary bile acids DCA and LCA exhibit concentration-dependent duality in intestinal barrier regulation. At physiological levels, DCA enhances mucosal defense by promoting MUC2 secretion and tight junction reinforcement (occludin/ZO-1). Conversely, recent research has indicated that supraphysiological DCA/LCA concentrations disrupt barrier integrity by activating TLR4/NLRP3 inflammasome stimulation in lamina propria macrophages.

It’s reported that AKK orchestrates a protective triad in atherosclerotic pathogenesis by synchronizing BA metabolism, intestinal barrier fortification, and immunometabolism regulation. We further analysed the connection between AKK, SBAs and the intestinal barrier protective effect of ZXYF. Previous studies suggested that AKK mitigates intestinal barrier injury by regulating microbial biotransformation of SBAs (DCA and LCA). Mechanically, AKK competitively inhibits 7α-dihydroxylation and reducing DCA/LCA overproduction, which prevents pathological DCA/LCA accumulation induced mitochondrial ROS and NLRP3 inflammasome activation in colonocytes. Here, integrated multi-modal analysis revealed significant positive associations between SBAs and intestinal barrier dysfunction biomarkers, while demonstrating inverse correlations with AKK abundance (Fig. 7A, B, D). This triad of evidence mechanistically links SBA accumulation to AKK-mediated barrier deterioration through microbial bile acid metabolism dysregulation.

The BA enterohepatic circuit exhibits > 95% recycling efficiency, with only ~ 5% spillover into the colon driving cholesterol catabolism [23]. Our findings illuminate FXR's dual regulatory role: (1) Hepatic FXR activation inhibits CYP7A1 to reduce de novo BA synthesis, and (2) Intestinal FXR engagement by endogenous ligands (CDCA/LCA/DCA) induces FGF15/19-mediated feedback inhibition of BA production [24]. Crucially, ZXYF treatment significantly lowered colonic SBAs through AKK enrichment. This microbial shift correlated with restored FXR signaling and improved barrier function, suggesting microbiota-FXR crosstalk mediates ZXYF's therapeutic effects. To investigate the regulatory effects of ZXYF on the enterohepatic circulation of bile acids, we conducted an analysis of the major components of ZXYF using UHPLC-O-Exactive-Orbitrap/MS mass spectrometry and subsequently performed molecular docking with FXR (Fig. 8A). The findings revealed that Alisol A and Alisol B exhibit strong binding affinity with FXR. Prior studies have shown that Alisol B 23-acetate, acting as an FXR agonist, can stimulate the FXR-BSEP pathway to increase excretion of fecal bile acid and cholesterol while decreasing plasma cholesterol levels [25]. To mechanistically dissect FXR-dependent pathways mediating ZXYF's effects on BA homeostasis and intestinal integrity, we implemented a bidirectional pharmacological strategy in FFA-stressed Caco-2/HepG2 cocultures. Cells were cotreated with ZXYF plus either the FXR-selective agonist fexaramine (5 μM) or antagonist GSK2033 (5 μM) (Fig. 9A, H). Intestinal compartment analysis revealed that FXR agonism completely abrogated ZXYF-induced FGF19 downregulation and barrier restoration. Conversely, hepatic FXR inhibition nullified ZXYF's lipid-lowering effects and BA regulatory capacity. This cellular compartment-specific response pattern definitively establishes FXR as the central molecular target governing ZXYF's dual therapeutic actions.

In this study, we observed that HFD suppressed hepatic FXR expression, in contrast to its effect on the in intestinal. Conversely, administration of ZXYF led to an increase in hepatic FXR expression (Fig. 8D). This phenomenon may be attributed to the enhanced emulsifying properties and increased fat absorption resulting from the binding of primary bile acids with taurine after synthesis in the liver. Tβ-MCA acid acts as an antagonist of FXR, and excessive lipid intake can stimulate the synthesis of Tβ-MCA, thereby inhibiting hepatic FXR expression. Above all, these results indicated that ZXYF accelerate hepatic excretion of cholesterol and synthesis of bile acids, while simultaneously inhibiting bile acids enterohepatic circulation to facilitate intestinal bile excretion through feces.

Our findings demonstrate that ZXYF alleviates lipid metabolic abnormalities in AS mice by restoring bile acid enterohepatic circulation via the FXR/FGF15 pathway, highlighting its therapeutic potential for metabolic disorders. Notably, 16S rDNA sequencing and metabolomic analyses revealed a concurrent increase in AKK abundance alongside improved bile acid homeostasis and lipid profiles. While these correlations suggest a plausible interplay between gut microbiota remodeling and the herb’s efficacy, we recognize that the current evidence does not establish direct causation or delineate whether AKK enrichment is a driver or consequence of FXR/FGF15 activation. Functional validation—such as fecal microbiota transplantation (FMT) or *Akkermansia* supplementation in FXR-deficient models—is essential to dissect whether microbial shifts are mechanistically linked to bile acid signaling or represent secondary effects of metabolic amelioration. Nevertheless, our data provide a robust foundation for understanding how herbal interventions modulate host-microbiota co-metabolism, with *Akkermansia* emerging as a promising biomarker and/or mediator warranting deeper mechanistic exploration. This study demonstrates that ZXYF ameliorates HFD-induced intestinal barrier dysfunction primarily by restoring bile acid enterohepatic circulation and reducing cytotoxic secondary bile acids (e.g., DCA, LCA). Nevertheless, we acknowledge that dissecting the interplay between bile acid dynamics, microbial metabolites, and host signaling pathways represents a critical future direction. Furthermore, TMAO has been confirmed as an independent predictor and accelerator of AS. TMAO is a substance metabolized by the gut microbiota, mainly derived from components like choline and L-carnitine in foods such as red meat, eggs, and milk. In our previous studies, it was found that the intervention of ZXYF could effectively reduce the level of TMAO. Recent studies have revealed that there is also a connection between the metabolism of TMAO and bile acid metabolism. Further research should be carried out to explore the influence of ZXYF on the interrelationship between TMAO and bile acid metabolism. This research is expected to provide novel ideas and advancements for the comprehensive treatment of atherosclerosis, thereby promoting the ongoing utilization and development of Traditional Chinese Medicine in managing cardiovascular disorders.

## Conclusion

**Fig. 10:**
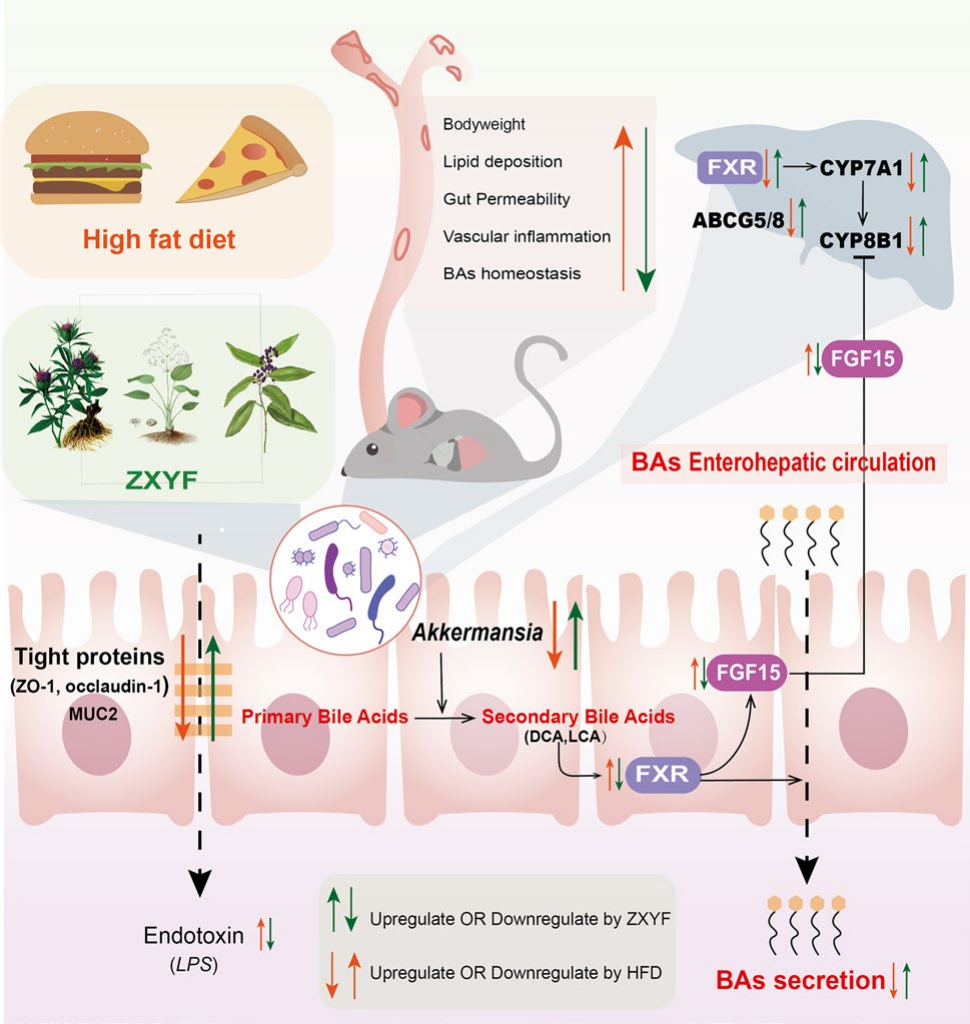
Proposed mechanism of ZXYF in ameliorating atherosclerosis via gut microbiota- BA axis modulation. This schematic integrates metagenomic and metabolomic analyses to illustrate how ZXYF intervention: (1) restores gut microbial homeostasis by enriching beneficial taxa (e.g.,*Akkermansia*); (2) enhances intestinal barrier integrity through upregulation of tight junction proteins (ZO-1, Occludin) and mucus layer components (MUC2) ; (3) modulates BA metabolism via FXR/FGF15 signaling-mediated regulation of hepatic CYP7A1; and (4) ultimately attenuates atherogenesis by reducing systemic inflammation (TNF-α, IL-6) and aortic plaque formation. Red arrows indicate pathological alterations in ApoE^−/−^ model, while green arrows denote ZXYF-induced therapeutic reversal

ZXYF were investigated in HFD-induced AS mice to determine its therapeutic effects on lipid disorder and atherosclerotic lesion. As shown in Fig. 10, our study demonstrates that ZXYF alleviated HFD caused intestinal barrier damage and cholesterol metabolism disorders by maintaining gut microbiota homeostasis and gut microbiome metabolism in ApoE^−/−^ mice. Our findings revealed that the metabolism of BAs was the key metabolic pathway involved in the effects of ZXYF. Moreover, ZXYF upregulated the liver BA synthesis and fecal BAs efflux in AS mice by activating the FXR/FGF15 signalling pathway in the hepatointestinal circulation of bile acids. This study highlights the potential of ZXYF for treating AS patients and provides novel insights into the critical role of intestinal barrier and BAs homeostasis for AS treatment.

## Supplementary Information

Additional file 1.

## Data Availability

The authors confirm that the data supporting the findings of this study are available within the article [and/or its supplementary materials].

## Abbreviations

ZXYF: ZeXieYin formula
AS: Atherosclerosis
BAs: Bile acids
ASV: Amplicon sequence variant
CYP7A1: Cholesterol 7α hydroxylase
DCA: Deoxycholic acid
LCA: Lithocholic acid
TCA: Taurocholic acid
PBAs: Primary bile acids
SBAs: Secondary bile acids
TDCA: Taurodeoxycholic acid
TNF-α: Tumour necrosis factor-α
α-MCA: α-Muricholic acid
