# Supplementary material for: ZeXieYin formula alleviates atherosclerosis by regulating SBAs levels through the FXR/FGF15 pathway and restoring intestinal barrier integrity
Source: Chin Med. 2025 May 25;20:68. doi: 10.1186/s13020-025-01116-y (PMC12103746; doi:10.1186/s13020-025-01116-y)
Supplement: Supplementary file 1 — Additional file 1. [file 13020_2025_1116_MOESM1_ESM.docx]

**Supplementary material**

**1.Materials and method**

- 1. ***Untargeted metabolomics study.***

**UHPLC-MS/MS analysis**

Metabolomics profiling was analyzed using a UPLC-ESI-Q-Orbitrap-MS system (UHPLC, Shimadzu Nexera X2 LC-30AD, Shimadzu, Japan) coupled with Q-Exactive Plus (Thermo Scientific, San Jose, USA).

For liquid chromatography (LC) separation, samples were analyzed using a ACQUITY UPLC® HSS T3 column （2.1×100 mm, 1.8μm）(Waters, Milford, MA, USA). The flow rate was 0.3 mL/min and the mobile phase contained: A: 0.1% FA in water and B: 100% acetonitrile (ACN). The gradient was 0% buffer B for 2 min and was linearly increase to 48% in 4 min, and then up to 100% in4 min and maintained for 2 min, and then decreased to 0% buffer B in 0.1 min, with 3 min re-equilibration period employed.

The electrospray ionization (ESI) with positive-mode and negative mode were applied for MS data acquisition separately. The HESI source conditions were set as follows: Spray Voltage：3.8kv (positive) and 3.2kv (negative)；Capillary Temperature：320 ℃; Sheath Gas (nitrogen) flow: 30 arb (arbitrary units); Aux Gas flow: 5 arb; Probe Heater Temp: 350 ℃; S-Lens RF Level：50. The instrument was set to acquire over the m/z range 70-1050 Da for full MS. The full MS scans were acquired at a resolution of 70,000 at m/z 200, and 17,500 at m/z 200 for MS/MS scan. The maximum injection time was set to for 100 ms for MS and 50 ms for MS/MS. The isolation window for MS2 was set to 2 m/z and the normalized collision energy (stepped) was set as 20, 30 and 40 for fragmentation.

**Data preprocessing and filtering**

The raw MS data were processed using MS-DIAL for peak alignment, retention time correction and peak area extraction. The metabolites were identified by accuracy mass (mass tolerance < 10 ppm) and MS/MS data (mass tolerance < 0.02Da) which were matched with HMDB, massbank and other public databases and our self-built metabolite standard library. In the extracted-ion features, only the variables having more than 50% of the nonzero measurement values in at least one group were kept.

**Multivariate statistical analysis**

R(version:4.0.3) and R packages were used for all multivariate data analyses and modeling. Data were mean-centered using Pareto scaling. Models were built on principal component analysis (PCA), orthogonal partial least-square discriminant analysis (PLS-DA) and partial least-square discriminant analysis (OPLS-DA). All the models evaluated were tested for over fitting with methods of permutation tests. The descriptive performance of the models was determined by R2X (cumulative) (perfect model: R2X (cum) = 1) and R2Y (cumulative) (perfect model: R2Y (cum) = 1) values while their prediction performance was measured by Q2 (cumulative) (perfect model: Q2 (cum) = 1) and a permutation test (n = 200). The permuted model should not be able to predict classes: R2 and Q2 values at the Y-axis intercept must be lower than those of Q2 and the R2 of the non-permuted model. OPLS-DA allowed the determination of discriminating metabolites using the variable importance on projection (VIP). The VIP score value indicates the contribution of a variable to the discrimination between all the classes of samples. Mathematically, these scores are calculated for each variable as a weighted sum of squares of PLS weights. The mean VIP value is 1, and usually VIP values over 1 are considered as significant. A high score is in agreement with a strong discriminatory ability and thus constitutes a criterion for the selection of biomarkers.

The discriminating metabolites were obtained using a statistically significant threshold of variable influence on projection (VIP) values obtained from the OPLS-DA model and two-tailed Student’s t test (p value) on the normalized raw data at univariate analysis level. The p value was calculated by one-way analysis of variance (ANOVA) for multiple groups analysis. Metabolites with VIP values greater than 1.0 and p value less than 0.05 were considered to be statistically significant metabolites. Fold change was calculated as the logarithm of the average mass response (area) ratio between two arbitrary classes. On the other side, the identified differential metabolites were used to perform cluster analyses with R package.

**KEGG Enrichment analysis**

To identify the perturbed biological pathways, the differential metabolite data were performed KEGG pathway analysis using KEGG database (http://www.kegg.jp). KEGG enrichment analyses were carried out with the Fisher’s exact test, and FDR correction for multiple testing was performed. Enriched KEGG pathways were nominally statistically significant at the p<0.05 level.

- 1. ***Targeted bile acid quantification.***

**Gas chromatography conditions:**

ACQUITY UPLC^®^ BEH C18 column (2.1×100 mm, 1.7μm, Waters, USA) was used, the injection volume was 5μL, the column temperature was 40℃, and the mobile phase was A-0.01% formic acid water, B-acetonitrile. The gradient elution conditions were 0~4 min, 25% B; 4~9 min, 25~30% B; 9~14 min, 30~36% B; 14~18 min, 36~38% B; 18~24 min, 38~50% B; 24~32 min, 50~75% B; 32~33 min, 75~90% B; 33~35.5 min, 90~25% B. The flow rate was 0.25 mL/min.

**Mass spectrum conditions:**

Electrospray ionization (ESI) source, negative ionization mode. The ion source temperature was 500℃, the ion source voltage was -4500 V, the collision gas was 6 psi, the curtain gas was 30 psi, and the atomizing gas and auxiliary gas were both 50 psi.Scans were performed using multiple reaction monitoring (MRM).

- 1. ***The list of primers used for real-time PCR.***

**Table S1**

The list of primers used for real-time PCR.

| **Gene name** | **Forward primer sequence (5′ to 3′)** | **Reverse primer sequence (5′ to 3′)** |
| --- | --- | --- |
| TNF-α | CCTGTAGCCCACGTCGTAG | GGGAGTAGACAAGGTACAACCC |
| IL-1β | GCAACTGTTCCTGAACTCAACT | ATCTTTTGGGGTCCGTCAACT |
| IL-6 | CTGCAAGAGACTTCCATCCAG | AGTGGTATAGACAGGTCTGTTGG |
| IL-10 | GCTCTTACTGACTGGCATGAG | CGCAGCTCTAGGAGCATGTG |
| FGFR4 | GTACCCTCGGACCGCGGCACATAC | GCCGAAGCTGCTGCCGTTGATG |
| BSEP | TCTGACTCAGTGATTCTTCGCA | GTGTAGAGTGAAGTCCTCCTTAGC |
| NTCPs | CAAACCTCAGAAGGACCAAACA | GTAGGAGGATTATTCCCGTTGTG |
| ABCG5 | TCAATGAGTTTTACGGCCTGAA | GCACATCGGGTGATTTAGCA |
| ABCG8 | TGCCCACCTTCCACATGTC | ATGAAGCCGGCAGTAAGGTAGA |
| ASBT | ACCACTTGCTCCACACTGCTT | CGTTCCTG AGTCAACCCACAT |
| OSTα | TGTTCCAGGTGCTTGTCATCC | CCACTGTTAGCCAAGATGGAGAA |
| OSTβ | GATGCGGCTCCTTGGAATTA | GGAGGAACATGCTTGTCATGAC |
| GAPDH | AGGTCGGTGTGAACGGATTTG | TGTAGACCATGTAGTTGAGGTCA |

**2. Results**

- 1. ***The fingerprint and the main components of ZXYF***

**Table S2**

Identification of the main components in ZXYF extract based on UPLC-Q-TOF-MS (Repeat times n=3, mg/g).

| Component | Batch | | | Average content |
| --- | --- | --- | --- | --- |
|  | 1 | 2 | 3 |  |
| Alisol A | 3.1124 | 3.2122 | 3.6670 | 3.3305 |
| Alisol B | 0.6102 | 0.2711 | 0.3663 | 0.4159 |
| Alisol C | 2.1567 | 2.5442 | 2.3456 | 2.3488 |
| Atractylenolide I | 1.0423 | 0.8842 | 0.9932 | 0.9732 |
| Atractylenolide II | 0.0676 | 0.0995 | 0.1124 | 0.0932 |
| Atractylenolide III | 0.1046 | 0.1123 | 0.1361 | 0.1177 |
| Monotropein | 0.7893 | 0.8765 | 0.8874 | 0.8511 |


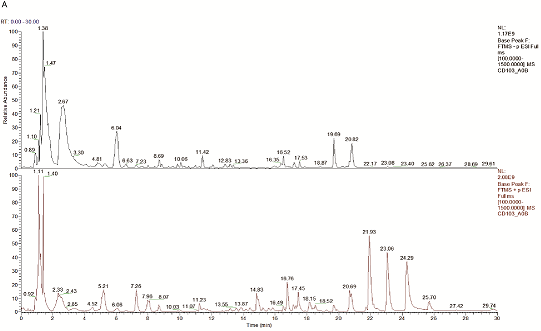
Fig.S1. Total ion flow chromatograms of ZXYF. The first column shows the total ion current plot in the negative ion mode in black, and the second column shows the total ion current plot in the positive ion mode in red.

- 1. ***The serum level of proinflammatory cytokines***

To determine the ZXYF effects on decreasing the vascular inflammatory response, we exam the serum levels of inflammatory cytokines associated with arteriosclerosis. As shown in Figure S2, ApoE^−/−^ mice consuming a HFD had markedly increased the levels of tumor necrosis factor-alpha (TNF-α), interleukin-1-beta (IL-1β), interleukin-10 (IL-10), and interleukin-6 (IL-6). LZXYF and HZXYF treatment significantly decreased the TNF-α, IL-1β, and IL-6 levels, but have no effects on the IL-10 level. These results indicate that, ZXYF treatment can relive vascular inflammation.


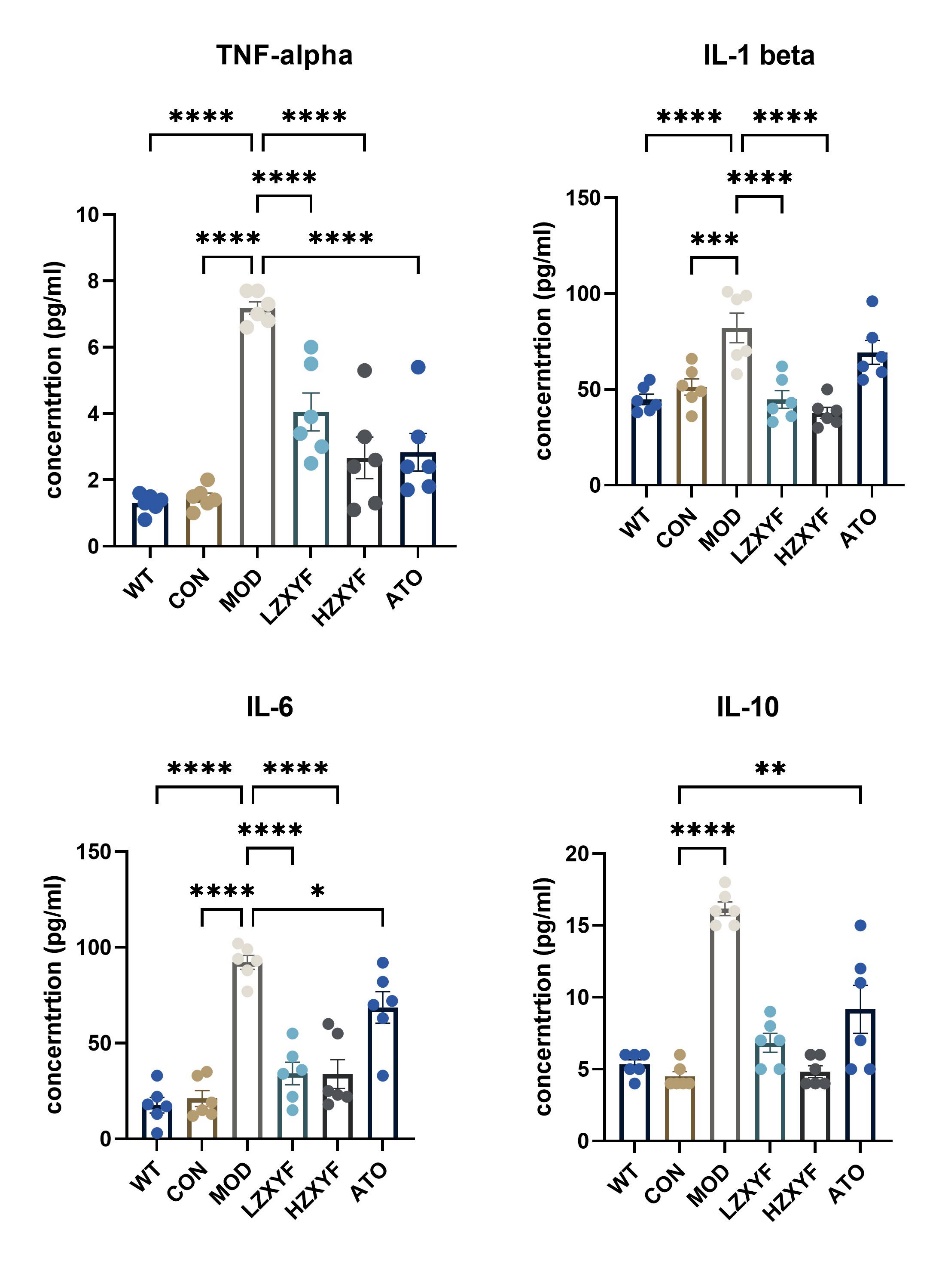


Fig.S2. The serum level of proinflammatory cytokine: TNF-α, IL-1β, IL-6 and IL-10. Data are expressed as mean ± standard deviation (SEM). One-way ANOVA was used to analyse statistical differences; **P* < 0.05, ***P* < 0.01, ****P* < 0.001

- 1. ***The CCK8 results and proinflammatory cytokines levels in Caco-2 cell.***


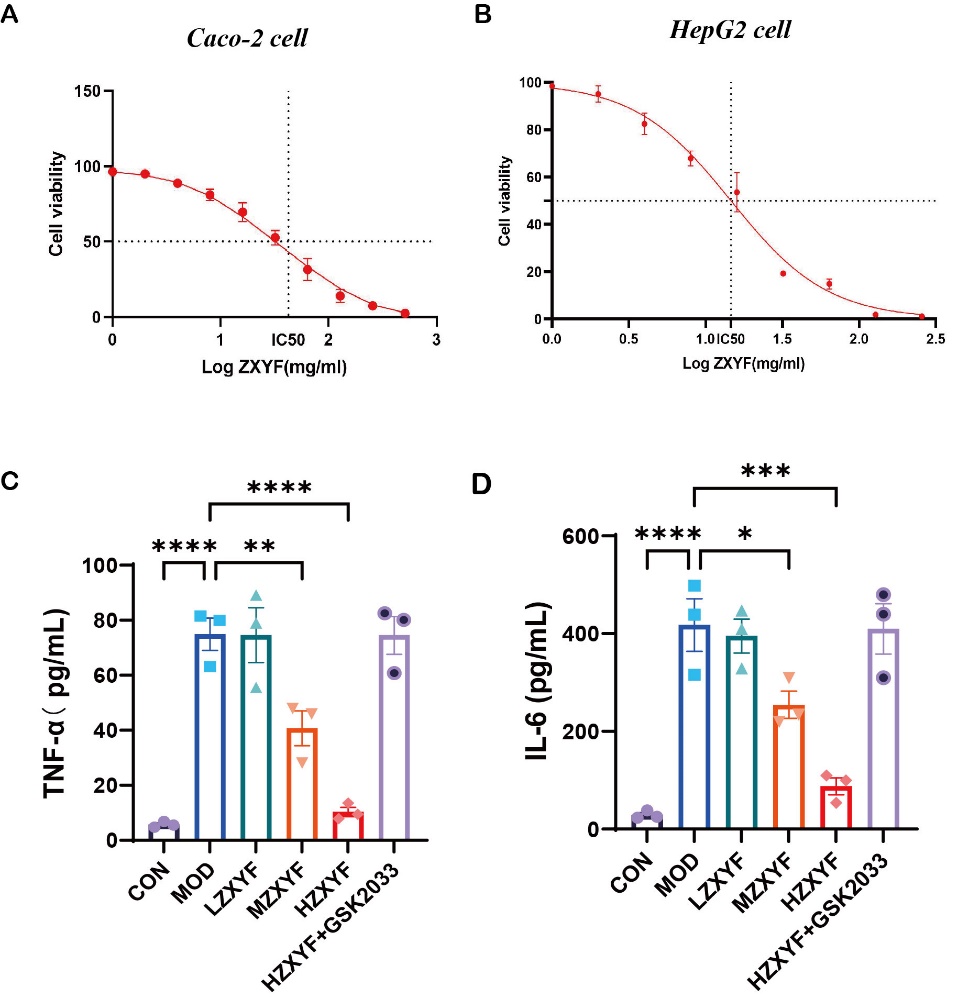
Prior to initiating experimental treatments, systematic cytotoxicity assessment using the Cell Counting Kit-8 (CCK-8) assay was performed to determine the non-toxic concentration range of ZXYF in both cellular models **(Figure S3A and B)**. These safety profiling data guided the selection of biologically relevant doses for downstream mechanistic studies. To elucidate the therapeutic potential of ZXYF in lipid-induced epithelial injury, we quantified TNF-α and IL-6 secretion patterns in Caco-2 monolayers exposed to FFA challenge. Both medium- and high-dose ZXYF (MZXYF/HZXYF) demonstrated significant anti-inflammatory efficacy, effectively mitigating FFA-triggered cytokine elevation **(Figure S3C, D)**. This dose-dependent suppression of proinflammatory mediators suggests ZXYF's capacity to restore intestinal barrier homeostasis.

Fig.S3. (A) The CCK8 results of ZXYF effect on Caco-2 cell. (B) The CCK8 results of ZXYF effect on HepG2 cell. (C) TNF-α and IL-6 levels in Caco-2 cells. Data are expressed as mean ± standard deviation (SEM). One-way ANOVA was used to analyse statistical differences; **P* < 0.05, ***P* < 0.01, ****P* < 0.001.

**Table S3**

The binding free energy of the major components in ZXYF was calculated through molecular docking.

| **Compounds** | **Receptor**  **(PDBID)** | **Binding free energy**  **(kcal/mol)** |
| --- | --- | --- |
| Aliso A | 1OSV | -10.2 |
| Aliso B | 1OSV | -10.5 |
| Aliso C | 1OSV | -9.9 |
| Atractylenolide_I | 1OSV | -9.1 |
| Atractylenolide_II | 1OSV | -9.2 |
| Atractylenolide_III | 1OSV | -9.2 |
| Monotropein | 1OSV | -7.8 |

***The original data of western blotting***


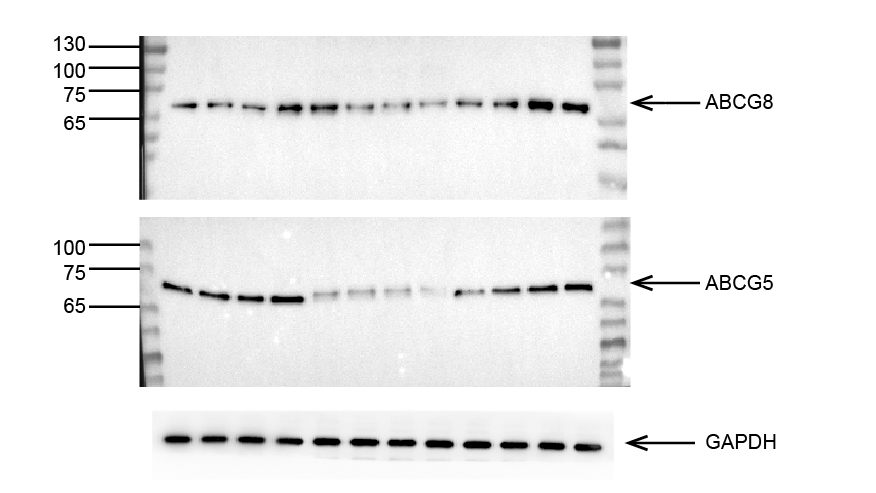


Fig.S4. Original western blot images of figure 6C.


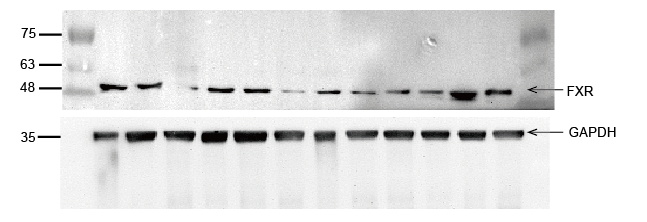


Fig.S5. Original western blot images of figure 8C.


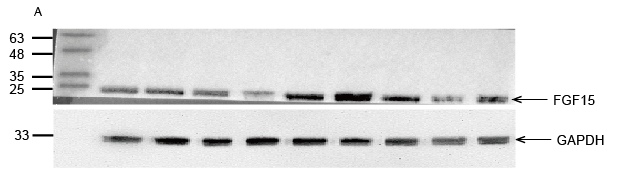


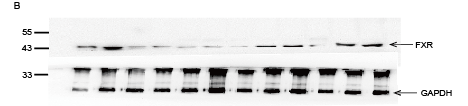


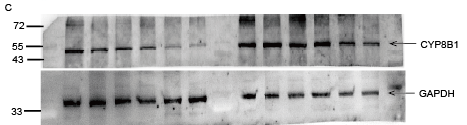


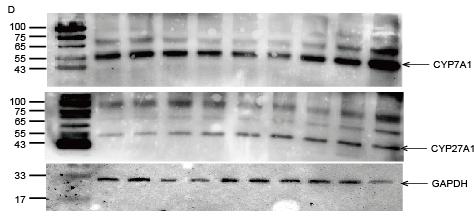


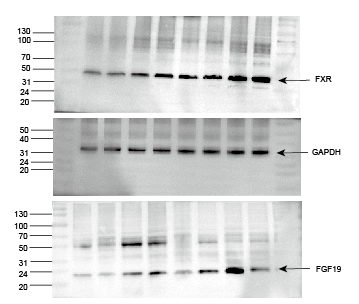
Fig.S6. Original western blot images of figure 8D and 8E.

Fig.S7. Original western blot images of figure 9D.


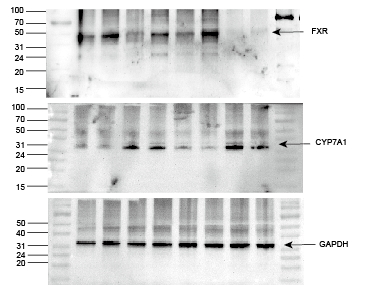
Fig.S8. Original western blot images of figure 9L.
